# Quantitative TaqMan^® ^real-time PCR assays for gene expression normalisation in feline tissues

**DOI:** 10.1186/1471-2199-10-106

**Published:** 2009-12-11

**Authors:** Yvonne Kessler, A Katrin Helfer-Hungerbuehler, Valentino Cattori, Marina L Meli, Bigna Zellweger, Pete Ossent, Barbara Riond, Claudia E Reusch, Hans Lutz, Regina Hofmann-Lehmann

**Affiliations:** 1 Clinical Laboratory, Vetsuisse Faculty, University of Zurich, Switzerland; 2 Institute of Veterinary Pathology, Vetsuisse Faculty, University of Zurich, Switzerland; 3 Clinic for Small Animal Internal Medicine, Vetsuisse Faculty, University of Zurich, Switzerland

## Abstract

**Background:**

Gene expression analysis is an important tool in contemporary research, with real-time PCR as the method of choice for quantifying transcription levels. Co-analysis of suitable reference genes is crucial for accurate expression normalisation. Reference gene expression may vary, e.g., among species or tissues; thus, candidate genes must be tested prior to use in expression studies. The domestic cat is an important study subject in both medical research and veterinary medicine. The aim of the present study was to develop TaqMan^® ^real-time PCR assays for eight potential reference genes and to test their applicability for feline samples, including blood, lymphoid, endocrine, and gastrointestinal tissues from healthy cats, and neoplastic tissues from FeLV-infected cats.

**Results:**

RNA extraction from tissues was optimised for minimal genomic DNA (gDNA) contamination without use of a DNase treatment. Real-time PCR assays were established and optimised for v-abl Abelson murine leukaemia viral oncogene homolog (ABL), β-actin (ACTB), β-2-microglobulin (B2M), β-glucuronidase (GUSB), hydroxymethyl-bilane synthase (HMBS), hypoxanthine phosphoribosyltransferase (HPRT), ribosomal protein S7 (RPS7), and tryptophan 5-monooxygenase activation protein, zeta polypeptide (YWHAZ). The presence of pseudogenes was confirmed for four of the eight investigated genes (ACTB, HPRT, RPS7, and YWHAZ). The assays were tested together with previously developed TaqMan^® ^assays for feline glyceraldehyde-3-phosphate dehydrogenase (GAPDH) and the universal 18S rRNA gene. Significant differences were found among the expression levels of the ten candidate reference genes, with a ~10^6^-fold expression difference between the most abundant (18S rRNA) and the least abundant genes (ABL, GUSB, and HMBS). The expression stability determined by the geNorm and NormFinder programs differed significantly. Using the ANOVA-based NormFinder program, RPS7 was the most stable gene in the tissues studied, followed by ACTB and ABL; B2M, HPRT, and the 18S rRNA genes were the least stable ones.

**Conclusion:**

The reference gene expression stability varied considerably among the feline tissues investigated. No tested gene was optimal for normalisation in all tissues. For the majority of the tissues, two to three reference genes were necessary for accurate normalisation. The present study yields essential information on the correct choice of feline reference genes depending on the tissues analysed.

## Background

The domestic cat is an important study subject not only in veterinary medicine but also in medical research. It plays an essential role as a laboratory model for human infectious, hereditary and endocrine diseases and allows the study of topics such as host-pathogen interactions, defence mechanisms, and development of prophylactic or therapeutic regimens. Of importance in this context is not only the feline immunodeficiency virus, the single naturally occurring animal model for HIV-AIDS pathogenesis [1,2] and the feline leukaemia virus (FeLV), an important model for retrovirus and tumour research [3,4], but also other infectious agents, some related to fatal human diseases [1,5]. Furthermore, in the genome of the cat, various mutations have been characterised that are associated with genetic diseases, and 280 phenes have been reported, 136 of which could potentially serve as models for human hereditary diseases http://omia.angis.org.au/. Models under investigation include the glycogen storage disease type IV reported in the Norwegian Forest cat, the only reported animal model for this pathology [6], or the obesity-associated form of diabetes mellitus in the domestic cat that is similar to the type 2 diabetes mellitus in humans [7]. For the latter, the domestic cat presents a valuable model for understanding the molecular mechanisms linking obesity to the development of insulin resistance, hypertension, and atherosclerosis [7]. The potential of the cat as an animal model and the similarities in genome organisation between humans and felids [8,9] provide the basis for a wide range of gene expression studies.

Quantitative real-time PCR assays are a method of choice for reliable and fast quantification of transcription levels in gene expression studies, and they are used frequently in many areas of modern research. Real-time PCR provides quantification of input templates over a broad linear range, low sample consumption, rapid throughput of large sample numbers, and low risk of contamination [10-12]. Accurate normalisation is of fundamental importance to obtaining sound results. Normalisation is usually achieved by simultaneous amplification of reference genes along with the target gene. Several publications emphasise the need for more than one reference gene for exact analysis of transcription levels [13-15]. When selecting reference genes, several critical points should be considered, including a stable, experimentally-independent expression pattern of the candidate gene, the absence of processed pseudogenes, an adequate level of expression, and a lack of potential co-regulation among target and reference genes [16-18].

For the domestic cat a number of potential reference genes have been studied using pair-wise correlation analysis (geNorm) [19] and real-time PCR systems based mainly on SYBR Green chemistry [13,20-22]. The SYBR Green principle has the advantage that it is less costly; however, TaqMan^® ^systems usually have a higher specificity and lead to less non-specific product formation than SYBR Green assays. No systematic study using TaqMan^® ^real-time PCR assays for potential feline reference genes is available. In addition, no comparisons of pair-wise analysis with ANOVA-based methods (NormFinder) [23] have been published, and most assays for feline reference genes were conducted using pathological samples. Data on tissues from healthy cats are largely missing.

Thus, the purpose of the present study was to i) develop and optimise TaqMan^® ^real-time PCR assays for potential feline reference genes and ii) evaluate the suitability of these assays for normalisation in the blood and other tissues from clinically healthy cats and neoplastic tissues. The earlier tissues were chosen to cover those frequently included in studies investigating infectious diseases and immunological, endocrine, metabolic, and inflammatory disorders. The neoplastic tissues originated from FeLV-infected cats; the expression of reference genes may differ in neoplastic tissues [16,24]. For stability comparison of the potential reference genes, two programs were used: the ANOVA-based NormFinder and the geNorm.

## Methods

### Sample collection

All domestic cats included in this study had been in experimental studies officially approved by the veterinary office of the appropriate Swiss Canton. They were kept in groups under optimal ethological conditions. Clinically healthy cats were available from negative control groups, and they were sacrificed for reasons unrelated to this study. Tissue samples were collected upon necropsy from 15 clinically healthy cats (ten neutered males and five intact females). The tissues were histologically examined and verified to be free of pathological alterations. They originated from lymphatic tissues including bone marrow (n = 11), mesenteric lymph node (n = 10), and spleen (n = 10); from the endocrine tissues of the adrenal gland (n = 11), pancreas (n = 13), thyroid (n = 10), and parathyroid (n = 7); from the gastrointestinal tissues of the parotid gland (n = 9), duodenum (n = 10), and ileum (n = 10); and from the brain (n = 13), myocardium (n = 10), kidney (n = 14), and liver (n = 9). The cats ranged in age from 1.25 to 13 years (median age 3.8 years). In addition, EDTA-anticoagulated whole blood samples were collected from 11 specific pathogen-free (SPF) cats (five males at the age of 0.5 years, five neutered males at 6 years, and two spayed females at 14 years). Upon necropsy, neoplastic tissues (n = 12, including tissue from liver (1), spleen (2), kidney (2), mesenteric lymph node (3), ileum (1), and thymus (3) were collected from six FeLV-infected cats (three neutered males, one neutered and two intact females; ages of 3 to 13 years). Five of the cats had been diagnosed with malignant lymphoma; one had leucosis. All tissues were snap-frozen upon collection and stored at -80°C until extraction of nucleic acids.

### Nucleic acid extractions

Tissues (30-35 mg, in duplicate) were homogenised prior to RNA extraction in 350 μl of RLT buffer (Qiagen, Hombrechtikon, Switzerland) containing 3.5 μl β-mercaptoethanol, together with a 5 mm Ø steel bead (Schieritz & Hauenstein, Arlesheim, Switzerland) in a Mixer Mill MM 300 (Retsch, Haan, Germany). Samples were then processed using the RNeasy Mini Kit (Qiagen) following the manufacturer's recommendations. In a preliminary experiment using selected samples (n = 10), the effect of a digestion step on the RNA binding silica gel membrane of the spin column, performed according to the manufacturer's instructions with RNase-free DNase, was assessed (on-column DNase treatment). In addition, for bone marrow, lymph node, spleen, and thyroid samples, the RNeasy Plus Mini Kit (Qiagen) with genomic DNA (gDNA) Eliminator spin columns and RLT plus buffer was applied according to the manufacturer's recommendations. The presence of contaminating gDNA was assessed using GAPDH quantitative reverse transcriptase (RT-) PCR with a minus-reverse transcription control with Reverse Transcriptase qPCR Mastermix (Eurogentec, Seraing, Belgium). RNA was extracted from 1 ml of blood within 60 minutes of collection using the QIAamp RNA Blood Mini Kit (Qiagen) and stored at -80°C until further use. gDNA was extracted from tissues using the DNeasy Blood & Tissue Kit (Qiagen). For all RNA and DNA extractions, negative controls consisting of 100 μl of phosphate buffered saline were prepared with each batch to monitor for cross-contamination.

### First-strand cDNA synthesis

The RNA yield and the ratio of absorbance at 260 nm to 280 nm (A_260_/A_280 _ratio) were measured using the NanoDrop ND-1000 Spectrophotometer (NanoDrop Technologies, Witec, Littau, Switzerland). Samples containing < 10 ng/μl of RNA were excluded from the study. First-strand cDNA was synthesised in quadruplicate using the High Capacity cDNA Reverse Transcription Kit (Applied Biosystems, Rotkreuz, Switzerland) and random primers according to the manufacturer's instructions. The amount of input RNA in each reaction was calculated to be 2 μg. The cDNA GAPDH copy number/RNA GAPDH copy number ratio was calculated as a measure of the efficiency of the cDNA synthesis; this ratio was used to normalise the reference gene copy numbers as assessed by quantitative real-time PCR.

### Development of real-time PCR assays for feline reference genes

Using Primer Express™ software (versions 2 and 3, Applied Biosystems), primers and TaqMan^® ^probes were designed for eight potential reference genes: ABL, ACTB, B2M, GUSB, HMBS, HPRT, RPS7, and YWHAZ (essential gene-specific data are given in Table 1). The sequences and information on gene organisation were retrieved from Ensembl http://www.ensembl.org/index.html, GenBank http://www.ncbi.nlm.nih.gov and the Genome Annotation Resource Fields (GARFIELD) http://lgd.abcc.ncifcrf.gov[25]. All systems were designed so that the predicted amplicons would span exon-exon boundaries (Table 2). The eight primer pairs (Microsynth, Balgach, Switzerland) were tested for amplification of the appropriate amplicon length using 5 μl of cDNA in a total volume of 25 μl per reaction on a Rotor-Gene 6000 real-time rotary analyser (Corbett, Mortlake, Australia) using the TaqMan^® ^Fast Universal PCR Master Mix (Applied Biosystems). Thermocycling conditions consisted of an initial denaturation of 20 s at 95°C, followed by 45 cycles of 95°C for 3 s and 60°C for 45 s. The PCR products were analysed by gel electrophoresis on 3% agarose gels and stained with ethidium bromide, and bands were visualised using the Chemigenius2 BioImaging System (Syngene, Cambridge, UK).

**Table 1:** Specifications of the tested potential feline reference genes

Gene	Name	Function	Accession Number
ABL	v-abl Abelson murine leukaemia viral oncogene homolog	Protein kinase; regulation of cell cycle, mismatch repair, DNA damage response	ENSFCAT00000005306^1^
ACTB	β-actin	Cytoskeletal structural protein	AB051104^2^
B2M	β-2-microglobulin	Major histocompatibility complex antigen class I receptor activity	NM_001009876^2^
GAPDH	Glyceraldehyde-3-phosphate dehydrogenase	Glycolytic enzyme	AF097177^2^
GUSB	β-glucuronidase	Glycoside hydrolase (carbohydrate metabolism)	NM_001009310^2^
HMBS	Hydroxymethyl-bilane synthase *Alias*: Porphobilinogen deaminase (PBGD)	Heme synthesis, porphyrin metabolism	ENSFCAG00000001160^1^
HPRT	Hypoxanthine phosphoribosyltransferase	Purine synthesis in salvage pathway	EF453697^2^
RPS7	Ribosomal protein S7	Ribosomal protein	NM_001009832^2^
YWHAZ	Tyrosine 3-monooxygenase *Alias*: tryptophan 5-monooxygenase activation protein, zeta polypeptide *Alias*: Phospholipase A2	Mediator of signal transduction	EF458621^2^
18S rRNA		Ribosomal RNA	X03205^2^

^1 ^Ensembl, (http://www.ensembl.org/index.html) ^2^GenBank

**Table 2:** Details of TaqMan^® ^real-time PCR assays

Gene	Oligo	Sequence	Amplicon size (bp)	Genomic position (exon)	Genomic (bp)	Detection gDNA	Pseudo-gene
ABL	Forward	TGTGGCGAGTGGTGATAATACAC	83	2	~12,000 ^7^	No	No
	Probe	CAGCATCACTAAAGGTGAAAAGCTACGAGTCCTT ^2^		2/3			
	Reverse	TCCACTCACCATTCTGGTTGTAA		3			
ACTB	Forward	CAACCGTGAGAAGATGACTCAGA	127	3/4	410 ^8^	(Yes) ^11^	Yes
	Probe	TCTCTGTACGCTTCTGGCCGCACC ^3^		4			
	Reverse	CCCAGAGTCCATGACAATACCA		4			
B2M	Forward	CGCGTTTTGTGGTCTTGGT	84	1	3,270 ^7^	No	No
	Probe	CGGACTGCTCTATCTGTCCCACCTGGA ^2^		1			
	Reverse	AAACCTGAACCTTTGGAGAATGC		1/2			
GAPDH	Forward	GCCATCAATGACCCCTTCAT	82	1	NA	(Yes) ^11,12^	Yes
	Probe	CTCAACTACATGGTCTACATGTTCCAGTATGATTCCA ^4^		1/2			
	Reverse	GCCGTGGAATTTGCCGT		2			
GUSB	Forward	CTACATCGATGACATCACCATCAG	80	4	532 ^9^	Yes	No
	Probe	ACCAGCGTGAACCAAGACACTGGGC ^3^		4/5			
	Reverse	CGCCTTCAACAAAAATCTGGTAA		5			
HMBS	Forward	TGGCAGTGCTGAAAGCCTTA	94	3	554 ^7^	No	No
	Probe	TTGAAATCGTTGCTATGTCCACCACAGG ^2^		3/4			
	Reverse	TTAGAGAGCGCAGTATCAAGAATCTT		4			
HPRT	Forward	AACTGGAAAGAATGTCTTGATTGTTG	100	4/5	>90,000 ^7^	(Yes) ^11^	Yes
	Probe	CACTGGCAAAACAATGCAAACCTTGCTTT ^3^		6			
	Reverse	GACCATCTTTGGATTATACTGCTTGA		6			
RPS7	Forward	GTCCCAGAAGCCGCACTTT	74	4/5	~2,200 ^10^	(Yes) ^11^	Yes
	Probe	CGCCGTGCACGACGCGA ^5^		5			
	Reverse	CACAATCTCGCTCGGGAAAA		5			
YWHAZ	Forward	ACAAAGACAGCACGCTAATAATGC	84	4	2,878 ^7^	(Yes) ^11^	Yes
	Probe	CGATGTCCACAATGTCAAGTTGTCTCTCAGTAAT ^3^		4/5			
	Reverse	CTTCAGCTTCATCTCCTTGGGTAT		5			
18S rRNA ^1^	Forward	CGGCTACCACATCCAAGGAA	NA	NA	NA	NT	NT
	Probe	TGCTGGCACCAGACTTGCCCTC ^6^		NA			
	Reverse	GCTGGAATTACCGCGGCT		NA			

^1 ^TaqMan^® ^Gene Expression Assay (Applied Biosystems); ^2 ^5' FAM/3' BHQ-1; ^3 ^5' Yakima Yellow/3' BHQ-1; ^4 ^5' FAM/3' TAMRA; ^5 ^5' TET/3' TAMRA; ^6 ^5' VIC/3' MGB; ^7 ^Ensembl; ^8 ^For ACTB, the PCR product was sequenced [GenBank: GQ848333]; it showed the highest identity to the human actin isoform gamma 1 [GenBank: NG_011433]. ^9 ^According to the Genome Annotation Resource Fields (GARFIELD) http://lgd.abcc.ncifcrf.gov[25]; ^10 ^According to Penning et al. [13]; ^11 ^Amplification of processed pseudogene; ^12 ^According to Leutenegger et al. [26]; NA = Not available; NT = Not tested

In order to test for potential amplification of pseudogenes or of gDNA, the eight primer pairs were also assayed with gDNA under the same conditions. Moreover, the possible presence of pseudogenes for the eight assays was assessed using the Ensembl Genome Browser. In all PCR assays, water was used as a negative control.

### Optimisation of quantitative real-time PCR assays

After the primers had been tested for correct amplification of the estimated amplicon length, the eight newly designed real-time TaqMan^® ^PCR systems were optimised using cDNA and a 3 × 3 primer matrix with 50, 300, and 900 nM end concentrations. Each of the nine conditions was run in quadruplicate under the conditions described above. Moreover, using the best primer concentration, five different probe (Microsynth) end concentrations (50, 100, 150, 200, and 250 nM) were tested for optimal performance. The optimised assays were tested together with a feline GAPDH TaqMan^® ^real-time PCR assay developed previously in our laboratory [26] and a universal 18S rRNA gene (Applied Biosystems).

### Production of DNA standards for absolute quantification

cDNA synthesised from feline tissue samples was used to generate standard templates for absolute quantification of ABL, ACTB, B2M, GUSB, HMBS, RPS7, and YWHAZ. The corresponding sequences were amplified using primers enclosing the TaqMan^® ^real-time PCR sequences under conditions described (Table 3) [27]. The gel purified amplification products (Gen Elute PCR Clean-Up Kit, Sigma-Aldrich, Buchs, Switzerland) were subjected to a 3' A-tailing reaction (Sigma) and ligated into the TOPO TA cloning vector pCRII (Invitrogen, Basel, Switzerland), selected by Ampicillin resistance, followed by sequencing (Microsynth). Plasmids were linearised by restriction digestion with *BamH*I (Promega, Wallisellen, Switzerland), *Spe*I (New England BioLabs, Berverly, MA, USA), or *Kpn*I (Roche, Rotkreuz, Switzerland) and then gel purified. The copy numbers were calculated based on spectrophotometric analysis (NanoDrop ND-1000). Carrier salmon sperm DNA (Invitrogen) at a concentration of 30 μg/ml was used for the tenfold serial dilutions of the standard templates, and aliquots of the dilutions were stored at -20°C until use. For the GAPDH assay, the DNA standard described previously [26] was used. For the HPRT and 18S rRNA assays, cDNA from kidney tissue of a clinically healthy cat was diluted tenfold in carrier salmon sperm DNA and in nuclease free water, respectively, to produce an arbitrary standard. The copy numbers of the latter samples were estimated by matching the resulting threshold cycle (Ct) values with those of the feline GAPDH standard.

**Table 3:** Primers used for production of standard templates

Gene	Forward Primer Reverse Primer	Sequences	Annealing Temperature (°C)	Amplification product (bp)
ABL	fABL_StdF	GGCTTTGAGGGAGACAAGAC	62	402
	fABL_StdR	GAAGCTGCCATTGATCAGAC		
ACTB	fACTB_StdF	CCATCGAACACGGCATTGTCAC	58	431
	fACTB_StdR	CTTGATGTCACGCACAATTTCCCG		
B2M	fB2M-F	GGCGCGTTTTGTGGTCTTGGTC	63	339
	fB2M-R	CACTTAACGACCTTGGGCTC		
GUSB	fGUSB_StdF	GCCGCATTACCATTGCCATCAAC	64	384
	fGUSB_StdR	GCATCAGGTATGGCCACCAGAG		
HMBS	fHMBS_StdF	CAGCCCAAAGATGAGAGTGATTCG	64	339
	fHMBS_StdR	GGGTGAAAGACAACGGCATCATAG		
RPS7	fRPS7-F	AGCTGAGGGAGCTGAACATC	65	432
	fRPS7-R	TGCCCGTGAGCTTCTTATAG		
YWHAZ	fYWHAZ_StdF	GAGGTTGCTGCTGGTGATGAC	64	329
	fYWHAZ_StdR	CCTGCTTCAGCTTCATCTCCTTGG		

### Efficiency, sensitivity, linear range and precision of the real-time PCR assays

The efficiency of the newly designed assays was calculated as described [28] using the following equation: E = (10^(-1/slope)^)-1. The sensitivity of the seven new systems (for which DNA standards had been produced) was determined by an endpoint dilution experiment: ten replicates of the dilutions containing 10^2^, 10^1^, and 10^0 ^standard template copies per reaction, respectively, were tested. The sensitivity of the assay is given by the dilution in which at least seven of 10 replicates are still positive [29]. The linear range of amplification and the precision of all newly developed TaqMan^® ^real-time PCR assays were determined using tenfold serial dilutions of the plasmid or arbitrary standards. For the precision analysis, the dilutions were chosen according to the ranges of Ct values that were characteristic for the expression levels of the particular reference genes in the tissues. Intra-run (n = 10) and between-run (n = 5) analytical performances of the PCR measurements were determined using these control materials.

### Data evaluation and statistics

For stability comparison of candidate reference genes, the Microsoft Excel Add-in NormFinder [23] was applied. Comparisons were made with calculations performed using the geNorm version 3.4 [19]. The NormFinder uses an ANOVA-based model [23], while the geNorm calculates the stability using a pairwise comparison model [19]. In addition, the geNorm ranks candidate reference genes according to the average expression stability, *M *[19]. Genes with the lowest *M *values have the most stable expression; a cut-off of 1.5 was proposed, above which the variation is assumed to be too high for accurate normalisation [19]. Moreover, the optimal number of reference genes required for accurate normalisation was estimated using the geNorm. To this end, the pairwise variation *V*_*n*/*n*+1 _between sequential normalisation factors containing an increasing number of reference genes was calculated. If *V*_*n*/*n*+1 _< 0.15, the recommended number of reference genes is given by n; the inclusion of an additional reference gene is not required [19]. Statistical analyses were performed with GraphPad Prism for Windows, Version 4.03 (GraphPad software, San Diego, CA). Expression levels of individual genes in different tissues were tested for statistical differences among several groups using the non-parametric Kruskal-Wallis test (pKW) and the Dunn's Multiple Comparison Test (pD). The expression levels of different genes in individual samples were tested for statistical differences between two groups using the non-parametric Wilcoxon signed rank test for paired samples (pW) and among several groups using the non-parametric Friedman test for paired samples (pF) and the Dunn's Multiple Comparison Test (pD). P-values < 0.05 were considered significantly different.

## Results

### RNA extractions and gDNA contamination

RNA extractions from blood using the QIAamp Blood Mini Kit and from tissues using the RNeasy Mini Kit yielded RNA with a low level of contaminating gDNA (< 1%), with the exceptions of bone marrow, lymph node, spleen, and thyroid. When RNA extraction was performed for these four tissues using the RNeasy Plus Mini Kit, contaminating gDNA levels were < 1%. These additionally processed RNA samples were used for analysis of these four tissues. No on-column DNase treatment was used in the main experiment because the loss of RNA due to DNase treatment was > 85%, as determined in a preliminary experiment (data not shown). RNA purity was estimated from A_260_/A_280 _ratio; this ratio ranged from 1.7 to 2.1.

### Evaluation of the primer pairs

When the primers were tested in a conventional PCR with cDNA, all assays yielded PCR products of the predicted size (Table 2). The primers were then assessed using the same procedure and gDNA to test for the amplification of gDNA and the possible presence of pseudogenes. Bands of the size of the cDNA were found for ACTB, HPRT, RPS7, and YWHAZ, indicating the presence of processed pseudogenes for these genes, but not for ABL, B2M, HMBS, and GUSB (Table 2). This was consistent with the results retrieved from Ensembl. In addition, for ACTB, GUSB, and HMBS, PCR products presumably of the size of the gDNA, including the introns, were detected (Table 2).

### Evaluation and optimisation of the newly developed real-time PCR assays

Primer and probe concentrations for the eight newly designed TaqMan^® ^real-time PCR assays were optimised using cDNA (for final concentrations see Table 4). When the real-time TaqMan^® ^PCR assays were tested using gDNA instead of cDNA, specific amplification was found for ACTB, HPRT, RPS7, and YWHAZ, confirming the presence of pseudogenes (Table 2). In addition, amplification was detected for GUSB (Table 2).

**Table 4:** Optimal final concentration of primers and probe for the newly designed real-time PCR assays

Real-time PCR assay	Forward Primer (nM)	Reverse Primer (nM)	Probe (nM)
ABL	300	900	50
ACTB	900	900	50
B2M	300	900	100
GUSB	900	300	100
HMBS	900	900	250
HPRT	900	900	50
RPS7	50	900	200
YWHAZ	900	900	150

### Efficiency, sensitivity, linear range and precision of the real-time PCR assays

The amplification efficiencies of the eight newly designed assays and the feline GAPDH real-time PCR were ≥96%. The lower detection limit of the assays for ABL, ACTB, B2M, GUSB, RPS7, and YWHAZ was equal to one copy of target standard plasmid per reaction in an endpoint dilution experiment (7 to 10 out of 10 reactions positive). For HMBS the lower limit of detection was < 100 copies per reaction (10 out of 10 reactions positive). For all newly developed TaqMan^® ^real-time PCR assays, we observed linearity of the assay over a ≥10^8^-fold range. The coefficients of variation ranged from 0.44% (B2M) to 1.18% (YWHAZ) for the intra-run precision analysis and from 0.49% (ACTB) to 2.15% (HMBS) for the between-run analysis.

### Expression levels of candidate reference genes

**Figure 1:**
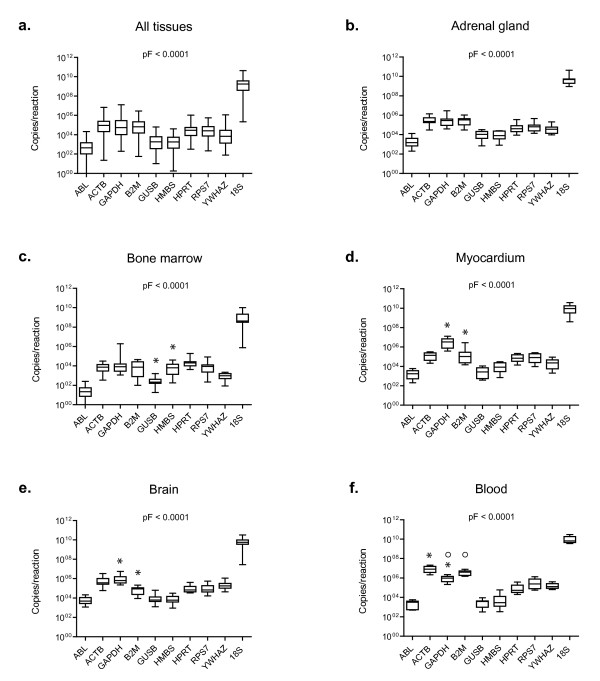
**Expression levels of candidate reference genes in different tissues**. a) All healthy tissues combined, b) adrenal gland, c) bone marrow, d) myocardium, e) brain, and f) blood samples. Values are given as copy numbers per PCR. For the 18S rRNA gene and HPRT the copy numbers were calculated using an arbitrary standard (see M&M). Data are shown as box plots. Boxes extend from the 25th to the 75th percentile; a horizontal line represents the median, and the error bars extend down to the smallest and up to the largest value. Expression levels were analyzed for statistical differences using the Friedman test (pF-values as indicated in the figure). Significant differences between two particular genes were analyzed by the Wilcoxon test for paired samples (asterisks and circles, respectively, mark statistically significant differences between two genes; pW ≤ 0.0039).

**Figure 2:**
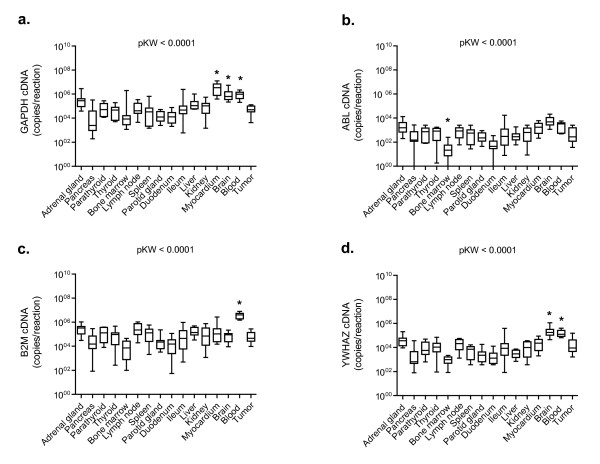
**Expression levels of selected reference genes in individual tissues**. a) GAPDH, b) ABL, c) B2M, and d) YWHAZ. Values are given as copy numbers per PCR. Data are shown as box plots. Boxes extend from the 25th to the 75th percentile; a horizontal line represents the median, and the error bars extend down to the smallest and up to the largest value. Expression levels were tested for statistical differences by the Kruskal-Wallis test (pKW-values as indicated in the figure) and the Dunn's post test (asterisks indicate statistically significant differences; pD < 0.001).

Transcription of the ten candidate reference genes was detectable above background in all tissue and blood samples from all cats tested. The potential reference genes were classified into three groups according to their transcription levels (all healthy tissues and blood samples were included in the analysis; Figure 1a). The difference in median expression levels was 10^6 ^between the most abundant and least abundant transcripts: 18S rRNA showed a high transcription level (median copy number/reaction ~10^9^); ACTB, GAPDH, B2M, HPRT, and RPS7 were found to have intermediate transcription levels (median copy number/reaction 2.6 × 10^4 ^to 9.3 × 10^4^), and ABL, GUSB, and HMBS had low transcription levels (median copy number/reaction 0.5 × 10^3 ^to 1.9 × 10^3^; Figure 1a). YWHAZ had a transcription level between the intermediate and low levels (median copy number/reaction 6.9 × 10^3^). Individual candidate reference genes had different expression levels across all studied tissues; the transcription levels differed significantly among all different reference genes when all tissues were included in the analysis (pF < 0.0001; pD < 0.05), with the exceptions of ACTB, GAPDH and B2M; GUSB and HMBS; and HPRT and RPS7 (Figure 1a). The latter three groups of reference genes did not have significantly different transcription levels (pD > 0.05; Figure 1a). A reference gene transcription level pattern similar to that seen in all tissues combined was found when individual tissues were examined (for a representative example, see Figure 1b), with the following particular exceptions. In the bone marrow samples HMBS transcription was significantly higher than GUSB transcription (pW = 0.0010; Figure 1c), in the myocardium and brain samples GAPDH transcription was significantly higher than B2M transcription (pW = 0.0020; Figure 1d and 1e), in the blood B2M and ACTB transcription levels were higher than GAPDH transcription (pW = 0.0039; Figure 1f), and in the liver YWHAZ was significantly lower than RPS7 and HPRT (pW = 0.0039, data not shown). When expression levels of the individual potential reference genes were analysed among different tissues significant differences were found. The most prominent were the following: GAPDH was significantly higher in the myocardium, brain, and blood samples than in most of the other tested tissues (pKW < 0.0001; pD < 0.001 for 11 of the other tested tissues; Figure 2a); ABL was significantly lower in the bone marrow samples than in the majority of the other tested tissues (pKW < 0.0001; pD < 0.001 for eight of the other tested tissues; Figure 2b), B2M was higher in the blood samples (pKW < 0.0001; pD < 0.001 for twelve of the other tested tissues; Figure 2c), and YWHAZ was higher in the blood and brain samples than in the majority of the other tissue samples (pKW < 0.0001; pD < 0.001 for 11 and 12, respectively, of the other tested tissues; Figure 2d). No particular differences in expression levels were observed when the neoplastic tissues were compared to the healthy tissues (pW > 0.05 for all genes tested; data not shown).

### Expression stability of candidate reference genes in different tissues

The stability of reference gene expression was estimated based on the calculations of the geNorm and NormFinder software, and the rank order given by the two programs differed significantly (Table 5; for details see also Additional Files 1 and 2). However, partial agreement was found between the NormFinder results and the results according to the *M *values of the geNorm program (Table 5); for three tissues (bone marrow, duodenum, and kidney) the rank order was identical. The two genes that ranked best were identical with both methods (manual *M *value ranking and NormFinder) for the majority of the tissues except for lymph node, parotid gland, liver, and blood samples. All further stability analyses were made using the NormFinder results.

**Table 5:** Ranking of potential reference genes according to the expression stability

Tissues	Analsysis	Ranking
All endocrine tissues tested	NF	YWHAZ > GUSB > B2M > RPS7> HMBS > ABL > 18S > GAPDH > ACTB > HPRT
	gN(M)	B2M > GUSB > YWHAZ > HMBS > RPS7 > ABL > 18S > GAPDH > ACTB > HPRT
	gN	B2M = HMBS > GUSB > YWHAZ > RPS7 > ABL > 18S > GAPDH > ACTB > HPRT
Adrenal gland	NF	HPRT > RPS7 > ABL > B2M > ACTB > GAPDH > 18S > HMBS > YWHAZ > GUSB
	gN(M)	HPRT > RPS7 > ABL > ACTB > B2M > 18S = GAPDH > HMBS > YWHAZ > GUSB
	gN	GAPDH = 18S > RPS7 > HPRT > YWHAZ > ABL > B2M > ACTB > HMBS > GUSB
Pancreas	NF	RPS7 > GUSB > YWHAZ > HMBS > B2M > GAPDH > ABL > 18S > HPRT > ACTB
	gN(M)	RPS7 > GUSB > YWHAZ > B2M > HMBS > ABL > GAPDH > 18S > HPRT > ACTB
	gN	GUSB = RPS7 > YWHAZ > HMBS > B2M > ABL > 18S > GAPDH > HPRT > ACTB
Parathyroid	NF	ACTB > RPS7 > GAPDH > HMBS > YWHAZ > ABL > B2M > GUSB > 18S > HPRT
	gN(M)	ACTB > RPS7 > GAPDH > ABL > HMBS > YWHAZ > B2M > GUSB > 18S > HPRT
	gN	GAPDH = RPS7 > ACTB > HMBS > ABL > YWHAZ > B2M > GUSB > 18S > HPRT
Thyroid	NF	GUSB > ACTB > YWHAZ > HMBS > B2M > RPS7 > ABL > 18S > GAPDH > HPRT
	gN(M)	ACTB > GUSB > YWHAZ > B2M > HMBS > RPS7 > ABL > 18S > GAPDH > HPRT
	gN	ACTB = HMBS > GUSB > B2M > YWHAZ > RPS7 > ABL > 18S > GAPDH > HPRT
All lymphoid tissues tested	NF	RPS7 > GUSB > ACTB > YWHAZ > ABL > GAPDH > B2M > HPRT > 18S > HMBS
	gN(M)	GUSB > RPS7 > ACTB > YWHAZ > ABL > B2M > GAPDH > HPRT > 18S > HMBS
	gN	GUSB = RPS7 > ACTB > YWHAZ > ABL > B2M > GAPDH > HPRT > 18S > HMBS
Bone marrow	NF	RPS7 > HMBS > ABL > GUSB > YWHAZ > ACTB > B2M > 18S > HPRT > GAPDH
	gN(M)	RPS7 > HMBS > ABL > GUSB > YWHAZ > ACTB > B2M > 18S > HPRT > GAPDH
	gN	ACTB = YWHAZ > HMBS > GUSB > RPS7 > ABL > B2M > 18S > HPRT > GAPDH
Lymph node	NF	GUSB > ACTB > B2M > HMBS > YWHAZ > ABL > RPS7 > GAPDH > 18S > HPRT
	gN(M)	GUSB > B2M > ACTB > YWHAZ > ABL > HMBS > RPS7 > GAPDH > 18S > HPRT
	gN	ABL = B2M > YWHAZ > ACTB > GUSB > HMBS > GAPDH > RPS7 > 18S > HPRT
Spleen	NF	GUSB > RPS7 > ACTB > ABL > B2M > HMBS > YWHAZ > HPRT > GAPDH > 18S
	gN(M)	GUSB > RPS7 > ACTB > B2M > ABL > HMBS > YWHAZ > HPRT > GAPDH > 18S
	gN	ACTB = B2M > GUSB > RPS7 > ABL > HMBS > YWHAZ > HPRT > GAPDH > 18S
All gastrointestinal tissues tested	NF	ACTB > HMBS > YWHAZ > RPS7 > ABL > GAPDH > GUSB > B2M > 18S > HPRT
	gN(M)	ACTB > HMBS > YWHAZ > RPS7 > ABL > GAPDH > GUSB > B2M > 18S > HPRT
	gN	ABL = HMBS > RPS7 > ACTB > YWHAZ > GAPDH > B2M > GUSB > 18S > HPRT
Parotid gland	NF	ABL > ACTB > RPS7 > YWHAZ > HMBS > B2M > HPRT > GAPDH > GUSB > 18S
	gN(M)	ABL > RPS7 > ACTB > YWHAZ > HMBS > B2M > HPRT > GAPDH > GUSB > 18S
	gN	ABL = ACTB > HMBS > RPS7 > YWHAZ > B2M > HPRT > GAPDH > GUSB > 18S
Duodenum	NF	RPS7 > GAPDH > ACTB > HMBS > YWHAZ > ABL > 18S > GUSB > B2M > HPRT
	gN(M)	RPS7 > GAPDH > ACTB > HMBS > YWHAZ > ABL > 18S > GUSB > B2M > HPRT
	gN	ACTB = HMBS > RPS7 > GAPDH > YWHAZ > ABL > 18S > GUSB > B2M > HPRT
Ileum	NF	HMBS > ABL > YWHAZ > ACTB > GAPDH > GUSB > RPS7 > HPRT > B2M > 18S
	gN(M)	HMBS > ABL > ACTB > YWHAZ > GAPDH > GUSB > RPS7 > HPRT > B2M > 18S
	gN	YWHAZ = ACTB > HMBS > ABL > GUSB > GAPDH > RPS7 > HPRT > B2M > 18S
Liver	NF	GUSB > GAPDH > RPS7 > HPRT > HMBS > YWHAZ > ABL > ACTB > B2M > 18S
	gN(M)	GAPDH > HMBS > HPRT > GUSB > RPS7 > YWHAZ > ABL > ACTB > B2M > 18S
	gN	ACTB = HMBS > ABL > GAPDH > HPRT > GUSB > YWHAZ > RPS7 > B2M > 18S
Kidney	NF	YWHAZ > RPS7 > ABL > ACTB > HMBS > GUSB > GAPDH > B2M > HPRT > 18S
	gN(M)	YWHAZ > RPS7 > ABL > ACTB > HMBS > GUSB > GAPDH > B2M > HPRT > 18S
	gN	RPS7 = YWHAZ > ABL > ACTB > HMBS > GAPDH > GUSB > B2M > HPRT > 18S
Myocardium	NF	ACTB > RPS7 > GAPDH > HMBS > YWHAZ > ABL > GUSB > 18S > HPRT > B2M
	gN(M)	RPS7 > ACTB > GAPDH > YWHAZ > HMBS > ABL > GUSB > 18S > HPRT > B2M
	gN	GAPDH = YWHAZ > RPS7 > ACTB > HMBS > ABL > GUSB > 18S > HPRT > B2M
Brain	NF	ACTB > ABL > RPS7 = YWHAZ > HMBS > GAPDH > HPRT > GUSB > B2M > 18S
	gN(M)	ACTB > ABL > RPS7 > YWHAZ > HPRT > GAPDH > HMBS > GUSB > B2M > 18S
	gN	ABL = RPS7 > ACTB > GAPDH > HPRT > YWHAZ > HMBS > GUSB > B2M > 18S
All healthy tissues combined	NF	RPS7 > GUSB > YWHAZ > ABL > ACTB > B2M > HMBS > 18S > GAPDH > HPRT
	gN(M)	RPS7 > GUSB > YWHAZ > ABL > ACTB > B2M > HMBS > 18S > GAPDH > HPRT
	gN	GUSB = RPS7 > ABL > YWHAZ > ACTB > B2M > HMBS > 18S > GAPDH > HPRT
Blood	NF	YWHAZ > ABL > GAPDH > B2M > ACTB > GUSB > 18S > HPRT > RPS7 > HMBS
	gN(M)	YWHAZ > B2M > GAPDH > ABL > ACTB > 18S > GUSB > HPRT > RPS7 > HMBS
	gN	YWHAZ = B2M > ACTB > 18S > GAPDH > ABL > GUSB > HPRT > RPS7 > HMBS
Neoplastic tissues	NF	RPS7 > ACTB > HMBS > GAPDH > B2M > ABL > 18S > GUSB > YWHAZ > HPRT
	gN(M)	ACTB > RPS7 > HMBS > GAPDH > B2M > 18S > ABL > GUSB > YWHAZ > HPRT
	gN	GAPDH = HMBS > 18S > RPS7 > ACTB > B2M > ABL > GUSB > YWHAZ > HPRT

The expression stability is given as stability values calculated by NormFinder (NF), *M *values calculated by geNorm (gN(M)), and geNorm calculations (gN). Rankings are shown from most (left) to least (right) stable genes.

The stability of the genes varied considerably depending on the tissues tested. When tissues were analysed individually, no single gene was found among the three best-ranked genes in all of the tested tissues. In 11 of the 14 healthy tissues, RPS7 ranked among the three most stable genes, followed by ACTB in eight and ABL in six out of 14 tissues (Table 5). Less stable were GUSB and YWHAZ; they were among the most stable genes in five of the 14 tissues used. GAPDH was among the three most stable genes in the NormFinder ranking in four out of 14 tissues. HMBS, B2M, HPRT, and 18S rRNA were the least stable, HMBS with two, B2M and HPRT with one, and 18S rRNA with no rankings among the most stable genes in the 14 tissues tested. When all 14 healthy tissues were included at once in the analysis (Table 5), RPS7, GUSB, and YWHAZ ranked as the best three reference genes, while ABL and ACTB ranked fourth and fifth. Exceptions were found in certain healthy tissues; RPS7 was less stable in the ileum, lymph node, and thyroid samples. Moreover, ACTB ranked last in the pancreas samples (Table 5). In the blood samples, GAPDH and B2M ranked better than in other tissues, but RPS7 ranked only second to last (Table 5). Remarkably, in the neoplastic tissues the two most stable genes were identical to those in all the healthy tissues (RPS7 and ACTB). ABL, GUSB, and YWHAZ were less stable than in the healthy tissues (Table 5).

### Number of reference genes for optimal normalization

The optimal number of reference genes for normalisation was calculated using the geNorm program. The number of recommended reference genes for optimal normalisation varied considerably depending on the tissue being tested. For brain, myocardium, lymph node, and adrenal gland the pairwise variation V_2/3 _was ≤ the proposed cut-off of 0.15 (Additional File 2); therefore, two reference genes should be sufficient for accurate normalisation in those tissues. Similarly, for parathyroid, parotid gland, liver and kidney, three; for thyroid, four; for spleen and neoplastic tissues, five; and for blood, seven reference genes were necessary for accurate normalisation according to the geNorm program (Additional File 2). For four tissues the pairwise variation *V *always exceeded the cut-off of 0.15. The recommended number of reference genes for these four tissues (lowest *V *value) was: five for the pancreas, six for ileum and bone marrow, and seven for the duodenum (Additional File 2). For all tissues combined, the optimal number of reference genes that would have been necessary for normalisation exceeded ten using the reference genes tested in this study. For the three tissue groups, endocrine, lymphatic, and gastrointestinal tissues, the *V *value always exceeded the cut-off of 0.15; the recommended number of reference genes for these tissue groups (lowest *V *value) was six (Additional File 2).

### Normalisation

When tissues were analysed individually, a normalisation factor was calculated for all tissues with the exception of the pancreas. For the latter, all *M *values calculated by geNorm exceeded the proposed cut-off value of 1.5, and no normalisation factors based on the geometric mean of multiple reference genes could be computed (Additional File 2). When tissue groups were analysed, a normalisation factor was calculated for the endocrine and gastrointestinal tissues but not for the lymphatic group. No normalisation factor was calculated for all tissues combined.

## Discussion

The present study is the first to develop and evaluate real-time TaqMan^® ^assays for the quantification of a series of potential feline reference genes. The assays were applied using feline peripheral blood and tissue samples from healthy cats. The latter were chosen based on their applicability in research areas such as the investigation of immune functions or metabolism, characterisation of infections, inflammatory reactions, or excretion patterns of pathogens. In this regard, the selected tissue categories also met the claim of suitability regarding their potential use in animal models for several human diseases. The expression stability of potential reference genes was analysed and compared for the feline species for the first time using pair-wise and ANOVA-based analyses; the two methods yielded discrepant results concerning the gene expression stability.

We selected ten commonly used mammalian reference genes featuring a broad range of cellular functions (Table 1). Some other potential candidate genes, such as Cyclophilin A, the TATA box binding protein or the transferring receptor CD71, were not included because they are regulated by or known to interact with retroviruses [30-33]; the latter are a major research interest of our group. Recently it was reported that nine of the ten selected genes, i.e., ACTB, ABL, B2M, GAPDH, GUSB, HMBS, HPRT, YWHAZ, and the 18S rRNA gene, are assumed not to be co-regulated [19,34,35].

Processed pseudogenes are known to hamper data interpretation in mRNA transcription analysis [36,37]. We therefore included potential reference genes that are known to lack pseudogenes in humans, i.e., ABL, B2M, GUSB, and HMBS [36,38]. We confirmed that these four genes also lack pseudogenes in the cat (at least for the sequences included in our assays). For the other four newly developed assays (ACTB, HPRT, RPS7, and YWHAZ), we demonstrated the presence of processed pseudogenes. For feline GAPDH the presence of one copy of a pseudogene had been reported earlier [39]. To minimise co-amplification of pseudogenes, gDNA contamination of the assayed RNA was reduced to a minimum by choosing the optimal RNA extraction method for each tissue. The inclusion of a DNase digestion step was omitted because it significantly reduced the RNA yield. The absence of a DNase treatment and thus the presence of sufficient amounts of RNA may explain why the PCR signals were clearly above background for all samples, in contrast to other reports in which HPRT, ABL, or HMBS could not (reliably) be detected [17,40].

We designed the eight new assays to span an exon-exon junction in order to reduce the possible interference of gDNA - including conventional but not processed pseudogenes. The assays for those four potential reference genes lacking processed pseudogenes did not amplify gDNA, with the exception of GUSB. For the latter, the amplicon size from gDNA was only 532 bp due to a short intron and the primer design software had positioned the exon-exon junction only three nucleotides before the 3' end of the probe (Table 2).

The expression level of an ideal reference gene should not undergo tissue-specific and experimentally-dependent variation and should be similar to that of the target genes [16-19,41,42]. The RNA transcription levels of the ten selected candidate reference genes in the blood samples and 15 tissues differed considerably, but most of them were within a range that could be used for proper normalisation of many target genes. Only the 18S rRNA gene expression level was impracticably high. Significant differences were found when the expression levels of the potential reference genes were analysed in different tissues. GAPDH expression was higher in brain, myocardium, and blood samples; this is consistent with results reported for human tissues [19,43]. B2M expression was significantly higher in blood. This has also been demonstrated for human leukocytes [19].

The expression stability of the tested reference genes was calculated using the NormFinder and the geNorm software. The geNorm uses a pairwise comparison model that provides a combination of two genes whose expressions are most correlated in the tested sample [19], while the ANOVA-based model of the NormFinder program selects the highest ranked gene based on the highest expression stability due to minimal estimated intra- and intergroup variation [23]. Using the geNorm, co-regulated genes may become highly ranked independent of their expression stabilities [23]. In contrast, the ANOVA-based approach is not significantly affected by co-regulation of candidate reference genes [23]. Therefore, the differences found between the reference gene rankings automatically produced by the two programs may indicate that at least some of the examined genes were co-regulated. A better agreement was found between the automatic NormFinder ranking and the ranking performed manually using the geNorm *M *values. To our knowledge, the differences observed between the automatically calculated geNorm gene ranking and the ranking using the *M *values of the geNorm program has not been reported previously.

Studies of the validation of reference gene expression have been performed for different mammalian species including humans [17,19,38,44], companion animals [13,14,45,46], farm animals [47-50], and horses [51,52]. No general best reference gene has been found for all species. Moreover and in agreement with findings from other species [17,41,42,53], in the cat no single gene was suitable for accurate normalisation in all investigated tissues. This highlights the need for proper validation of reference genes in the respective tissues preceding any experimental set-up. It has been demonstrated that normalisation of data sets with different reference genes, such as GAPDH and ACTB, may influence the outcome of the study; the expression profiles of target genes were markedly influenced and statistically significant differences between study groups were present or absent depending on the choice of the reference genes [54-56].

For the majority of the examined feline healthy tissues RPS7, ACTB, and ABL were among the most stably expressed genes. When all healthy tissues were combined for analysis, the three most stable genes were RPS7, GUSB, and YWHAZ. These results are only partially in agreement with those of a recent validation of feline reference genes using SYBR Green real-time PCR assays and pairwise analysis [13]. In the latter study, RPS7 was also found as the most stable gene in six tissues under investigation; YWHAZ ranked fourth (after two ribosomal protein genes not included in the present study), and GUSB was found among the least stable genes; ACTB and ABL were not examined [13]. Three feline tissues were included in both the previous [13] and the present study. While for the kidney a partial agreement was found in the gene ranking (RPS7 and YWHAZ were among the stable ones; HPRT and GAPDH were rather unstable), the results for liver and myocardium differed significantly. The observed discrepancy may be (apart from the differences in the applied PCR and mathematical methods) due to the tissues under investigation. We based our evaluation primarily on tissues from healthy cats. In contrast, the study from Penning and co-workers [13] used mainly pathological tissues. Pathological processes can alter the expression levels of reference genes [55,57], therefore it is of importance to evaluate potential reference genes in healthy tissues. Generally, healthy tissues are included in most study design as internal controls. Thus, with the present study, we provide baseline data on reference genes for many future studies investigating feline tissues. Furthermore, the number of samples studied may have influenced the evaluation: in the previous study, for the majority of the tissues, a small number of identical samples (i.e. kidney: n = 2 healthy tissue; n = 3 tumours; n = 3 chronic kidney failure) was available [13].

Although in the present study B2M in general was not a very stable reference gene in the tissues, it ranked second to fourth in stability in the blood samples. This would confirm a finding in human blood cells, where B2M seemed a good choice for normalisation in leukocytes, while it was one of the least stable genes in tissues [19]. For the neoplastic tissues, only a limited number of samples from FeLV-infected cats with malignant lymphoma and leucosis were available. The gene ranking order but not the expression levels of the reference genes in the neoplastic tissues differed significantly from those in the healthy tissues. Nonetheless, the two most stable genes in the neoplastic tissues (RPS7 and ACTB) were identical to those in the investigated healthy tissues. Thus, these two genes could serve as a good first choice to be tested in neoplastic tissues in future studies.

Using the geNorm program, we calculated that two to three reference genes were required for accurate normalisation in most tissues. According to Vandesompele and co-workers, using the three best reference genes is a valid normalisation strategy in most cases; it results in much more accurate and reliable normalisation than the use of only one reference gene does [19]. The use of a limited number of reference genes is also supported by the finding that no significant effect on the relative quantity of the target gene expression was demonstrated when using the combination of the two best genes compared to using five of the six most stable genes [34].

From our results, we recommend consulting the literature for appropriate reference genes according to the tissues and species under investigation. If healthy tissues are included in the study design (e.g. baseline values, negative controls) the reference genes should be evaluated in healthy individuals. If the corresponding data is not available, preliminary experiments for the identification of optimal reference genes are necessary. If this is impossible, we recommend using RPS7 as the primary choice for a reference gene in feline tissues (with tissue-specific limitations documented in the present study); results should be confirmed using other reference genes, such as ACTB, ABL, GUSB, and/or YWHAZ.

## Conclusions

In this study, we investigated the most reliable feline reference genes for normalisation of gene expression data in blood samples and 15 different tissues, including endocrine, lymphatic, and gastrointestinal tissues, using real-time TaqMan^® ^PCR assays and pair-wise and ANOVA-based analyses. Our results indicated that stability of the reference genes varies among the different tissues, and no gene was found among the most stable ones for all the tissues under investigation. Moreover, significant differences were found using either pair-wise or ANOVA-based analysis approaches. The three most stable genes were RPS7, ACTB, and ABL, while B2M, HPRT, and the 18S rRNA gene were the least stable ones. The frequently used GAPDH had an acceptable stability in a few tissues. These data emphasise the need for proper validation of candidate reference genes in the respective tissues and species in healthy individuals preceding the initiation of any experimental gene expression study.

## Abbreviations

A: absorbance; ABL: v-abl Abelson murine leukaemia viral oncogene homolog; ACTB: β-actin; B2M: β-2-microglobulin; Ct value: threshold cycle value; FeLV: feline leukaemia virus; GAPDH: glyceraldehyde-3-phosphate dehydrogenase; GUSB: β-glucuronidase; HMBS: hydroxymethyl-bilane synthase; HPRT: hypoxanthine phosphoribosyltransferase; *M *value: average expression stability; RPS7: ribosomal protein S7; RT-PCR: reverse transcriptase polymerase chain reaction; *V *value: variation of expression stability; *V*_*n*/*n*+1_: pairwise variation; YWHAZ: tyrosine 3-monooxygenase/tryptophan 5-monooxygenase activation protein, zeta polypeptide.

## Authors' contributions

YK performed and analysed the research and drafted the manuscript. AKH designed the assays, performed statistical analyses, prepared the figures, and co-drafted the manuscript. VC and MLM participated in the assay design and data analysis and revised the manuscript. BZ performed molecular assays. PO performed the necropsies, the macroscopic and histological analysis, and the sample collections and revised the manuscript. BR was responsible for the SPF cats, housing, animal care, and the blood collections and revised the manuscript. CER contributed to the study design (endocrine tissues) and provided some feline tissues. HL contributed to the study design. RHL conceived and supervised the study, participated in the data analyses, and edited the manuscript. All authors read and approved the final manuscript.

## Supplementary Material

Additional file 1 **NormFinder output**. Stability values and standard errors calculated by the NormFinder program. In addition, a gene ranking for every tissue is shown. Click here for file

Additional file 2 **geNorm output files**. Average expression stability values calculated by the geNorm program for reference genes included in this study and graph showing the optimal number of reference genes for normalization. Output files are shown for a) adrenal gland, b) pancreas, c) parathyroid, d) thyroid, e) bone marrow, f) lymph node, g) spleen, h) parotid gland, i) duodenum, j) ileum, k) liver, l) kidney, m) myocardium, n) brain, o) blood, p) neoplastic tissues, q) all 14 healthy tissues combined, r) all endocrine tissues, s) all lymphoid tissues, and t) all gastrointestinal tissues. Click here for file

## References

[B1] O'Brien SJ Menotti-Raymond M Murphy WJ Yuhki N The Feline Genome Project Annual Review of Genetics 2002 36 657 686 10.1146/annurev.genet.36.060602.145553 12359739

[B2] Elder JH Sundstrom M de Rozieres S de Parseval A Grant CK Lin YC Molecular mechanisms of FIV infection Veterinary Immunology and Immunopathology 2008 123 3 13 10.1016/j.vetimm.2008.01.007 18289701 PMC2409060

[B3] Onions D Animal models: lessons from feline and bovine leukaemia virus infections Leukemia Research 1985 9 709 711 10.1016/0145-2126(85)90280-2 2989623

[B4] Miyazawa T Infections of feline leukemia virus and feline immunodeficiency virus Frontiers in Bioscience 2002 7 d504 518 10.2741/miyazawa 11815291

[B5] Appel M Virus Infections of Carnivores 1987 Amsterdam: Elsevier Science Inc

[B6] Fyfe JC Giger U Van Winkle TJ Haskins ME Steinberg SA Wang P Patterson DF Glycogen storage disease type IV: inherited deficiency of branching enzyme activity in cats Pediatric Research 1992 32 719 725 10.1203/00006450-199212000-00020 1337588

[B7] Zini E Linscheid P Franchini M Kaufmann K Monnais E Kutter AP Ackermann M Lutz TA Reusch CE Partial sequencing and expression of genes involved in glucose metabolism in adipose tissues and skeletal muscle of healthy cats Veterinary Journal 2009 180 66 70 10.1016/j.tvjl.2007.10.022 18078768

[B8] O'Brien SJ Menotti-Raymond M Murphy WJ Nash WG Wienberg J Stanyon R Copeland NG Jenkins NA Womack JE Marshall Graves JA The promise of comparative genomics in mammals Science 1999 286 458 462 479-481 10.1126/science.286.5439.458 10521336

[B9] Murphy WJ Stanyon R O'Brien SJ Evolution of mammalian genome organization inferred from comparative gene mapping Genome Biology 2001 2 reviews0005.1 0005.8 10.1186/gb-2001-2-6-reviews0005 PMC138942 11423011

[B10] Gibson UE Heid CA Williams PM A novel method for real time quantitative RT-PCR Genome Research 1996 6 995 1001 10.1101/gr.6.10.995 8908519

[B11] Heid CA Stevens J Livak KJ Williams PM Real time quantitative PCR Genome Research 1996 6 986 994 10.1101/gr.6.10.986 8908518

[B12] Livak KJ Flood SJ Marmaro J Giusti W Deetz K Oligonucleotides with fluorescent dyes at opposite ends provide a quenched probe system useful for detecting PCR product and nucleic acid hybridization PCR Methods and Applications 1995 4 357 362 7580930 10.1101/gr.4.6.357

[B13] Penning LC Vrieling HE Brinkhof B Riemers FM Rothuizen J Rutteman GR Hazewinkel HA A validation of 10 feline reference genes for gene expression measurements in snap-frozen tissues Veterinary Immunology and Immunopathology 2007 120 212 222 10.1016/j.vetimm.2007.08.006 17904230

[B14] Peters IR Peeters D Helps CR Day MJ Development and application of multiple internal reference (housekeeper) gene assays for accurate normalisation of canine gene expression studies Veterinary Immunology and Immunopathology 2007 117 55 66 10.1016/j.vetimm.2007.01.011 17346803

[B15] Tricarico C Pinzani P Bianchi S Paglierani M Distante V Pazzagli M Bustin SA Orlando C Quantitative real-time reverse transcription polymerase chain reaction: normalization to rRNA or single housekeeping genes is inappropriate for human tissue biopsies Analytical Biochemistry 2002 309 293 300 10.1016/S0003-2697(02)00311-1 12413463

[B16] Jung M Ramankulov A Roigas J Johannsen M Ringsdorf M Kristiansen G Jung K In search of suitable reference genes for gene expression studies of human renal cell carcinoma by real-time PCR BMC Molecular Biology 2007 8 47 10.1186/1471-2199-8-47 17559644 PMC1913536

[B17] Radonic A Thulke S Mackay IM Landt O Siegert W Nitsche A Guideline to reference gene selection for quantitative real-time PCR Biochemical and Biophysical Research Communications 2004 313 856 862 10.1016/j.bbrc.2003.11.177 14706621

[B18] Schmittgen TD Zakrajsek BA Effect of experimental treatment on housekeeping gene expression: validation by real-time, quantitative RT-PCR Journal of Biochemical and Biophysical Methods 2000 46 69 81 10.1016/S0165-022X(00)00129-9 11086195

[B19] Vandesompele J De Preter K Pattyn F Poppe B Van Roy N De Paepe A Speleman F Accurate normalization of real-time quantitative RT-PCR data by geometric averaging of multiple internal control genes Genome Biology 2002 3 research0034.1 0034.11 10.1186/gb-2002-3-7-research0034 PMC126239 12184808

[B20] De Maria R Olivero M Iussich S Nakaichi M Murata T Biolatti B Di Renzo MF Spontaneous feline mammary carcinoma is a model of HER2 overexpressing poor prognosis human breast cancer Cancer Research 2005 65 907 912 15705889

[B21] Kobayashi S Sato R Inanami O Yamamori T Yamato O Maede Y Sato J Kuwabara M Naito Y Reduction of concanavalin A-induced expression of interferon-gamma by bovine lactoferrin in feline peripheral blood mononuclear cells Veterinary Immunology and Immunopathology 2005 105 75 84 10.1016/j.vetimm.2004.12.016 15797477

[B22] Koyama T Omata Y Maki Y Toyoda Y Saito A Interleukin-12, interferon-gamma and interleukin-4 gene expression in cats infected with Toxoplasma gondii Journal of Veterinary Medical Science 1999 61 819 821 10.1292/jvms.61.819 10458106

[B23] Andersen CL Jensen JL Orntoft TF Normalization of real-time quantitative reverse transcription-PCR data: a model-based variance estimation approach to identify genes suited for normalization, applied to bladder and colon cancer data sets Cancer Research 2004 64 5245 5250 10.1158/0008-5472.CAN-04-0496 15289330

[B24] Laidlaw AM Copeland B Ross CM Hardingham JE Extent of over-expression of hepatocyte growth factor receptor in colorectal tumours is dependent on the choice of normaliser Biochemical and Biophysical Research Communications 2006 341 1017 1021 10.1016/j.bbrc.2006.01.060 16458257

[B25] Pontius JU O'Brien SJ Genome Annotation Resource Fields--GARFIELD: a genome browser for Felis catus Journal of Heredity 2007 98 386 389 10.1093/jhered/esm055 17646276

[B26] Leutenegger CM Mislin CN Sigrist B Ehrengruber MU Hofmann-Lehmann R Lutz H Quantitative real-time PCR for the measurement of feline cytokine mRNA Veterinary Immunology and Immunopathology 1999 71 291 305 10.1016/S0165-2427(99)00100-2 10587308 PMC7119904

[B27] Willi B Boretti FS Baumgartner C Tasker S Wenger B Cattori V Meli ML Reusch CE Lutz H Hofmann-Lehmann R Prevalence, risk factor analysis, and follow-up of infections caused by three feline hemoplasma species in cats in Switzerland Journal of Clinical Microbiology 2006 44 961 969 10.1128/JCM.44.3.961-969.2006 16517884 PMC1393118

[B28] Klein D Janda P Steinborn R Muller M Salmons B Gunzburg WH Proviral load determination of different feline immunodeficiency virus isolates using real-time polymerase chain reaction: influence of mismatches on quantification Electrophoresis 1999 20 291 299 10.1002/(SICI)1522-2683(19990201)20:2<291::AID-ELPS291>3.0.CO;2-R 10197436

[B29] Lockey C Otto E Long Z Real-time fluorescence detection of a single DNA molecule Biotechniques 1998 24 744 746 9591119 10.2144/98245bm09

[B30] Savarino A Calosso L Piragino A Martini C Gennero L Pescarmona GP Pugliese A Modulation of surface transferrin receptors in lymphoid cells de novo infected with human immunodeficiency virus type-1 Cell Biochemistry and Function 1999 17 47 55 10.1002/(SICI)1099-0844(199903)17:1<47::AID-CBF810>3.0.CO;2-V 10191508

[B31] Towers GJ The control of viral infection by tripartite motif proteins and cyclophilin A Retrovirology 2007 4 40 10.1186/1742-4690-4-40 17565686 PMC1906832

[B32] Ylinen LM Schaller T Price A Fletcher AJ Noursadeghi M James LC Towers GJ Cyclophilin A levels dictate infection efficiency of human immunodeficiency virus type 1 capsid escape mutants A92E and G94D Journal of Virology 2009 83 2044 2047 10.1128/JVI.01876-08 19073742 PMC2643744

[B33] Brady J Kashanchi F Tat gets the "green" light on transcription initiation Retrovirology 2005 2 69 10.1186/1742-4690-2-69 16280076 PMC1308864

[B34] McNeill RE Miller N Kerin MJ Evaluation and validation of candidate endogenous control genes for real-time quantitative PCR studies of breast cancer BMC Molecular Biology 2007 8 107 10.1186/1471-2199-8-107 18042273 PMC2211316

[B35] He JQ Sandford AJ Wang IM Stepaniants S Knight DA Kicic A Stick SM Pare PD Selection of housekeeping genes for real-time PCR in atopic human bronchial epithelial cells European Respiratory Journal 2008 32 755 762 10.1183/09031936.00129107 18417509

[B36] Zhang Z Carriero N Gerstein M Comparative analysis of processed pseudogenes in the mouse and human genomes Trends in Genetics 2004 20 62 67 10.1016/j.tig.2003.12.005 14746985

[B37] Garbay B Boue-Grabot E Garret M Processed pseudogenes interfere with reverse transcriptase-polymerase chain reaction controls Analytical Biochemistry 1996 237 157 159 10.1006/abio.1996.0218 8660555

[B38] Lion T Current recommendations for positive controls in RT-PCR assays Leukemia 2001 15 1033 1037 10.1038/sj.leu.2402133 11455970

[B39] Molia S Chomel BB Kasten RW Leutenegger CM Steele BR Marker L Martenson JS Keet DF Bengis RG Peterson RP Prevalence of Bartonella infection in wild African lions (Panthera leo) and cheetahs (Acinonyx jubatus) Veterinary Microbiology 2004 100 31 41 10.1016/j.vetmic.2004.01.007 15135511

[B40] Zhang X Ding L Sandford AJ Selection of reference genes for gene expression studies in human neutrophils by real-time PCR BMC Molecular Biology 2005 6 4 10.1186/1471-2199-6-4 15720708 PMC551605

[B41] Bustin SA Absolute quantification of mRNA using real-time reverse transcription polymerase chain reaction assays Journal of Molecular Endocrinology 2000 25 169 193 10.1677/jme.0.0250169 11013345

[B42] Thellin O Zorzi W Lakaye B De Borman B Coumans B Hennen G Grisar T Igout A Heinen E Housekeeping genes as internal standards: use and limits Journal of Biotechnology 1999 75 291 295 10.1016/S0168-1656(99)00163-7 10617337

[B43] Barber RD Harmer DW Coleman RA Clark BJ GAPDH as a housekeeping gene: analysis of GAPDH mRNA expression in a panel of 72 human tissues Physiological Genomics 2005 21 389 395 10.1152/physiolgenomics.00025.2005 15769908

[B44] Beillard E Pallisgaard N Velden VH van der Bi W Dee R Schoot E van der Delabesse E Macintyre E Gottardi E Saglio G Evaluation of candidate control genes for diagnosis and residual disease detection in leukemic patients using 'real-time' quantitative reverse-transcriptase polymerase chain reaction (RQ-PCR) - a Europe against cancer program Leukemia 2003 17 2474 2486 10.1038/sj.leu.2403136 14562124

[B45] Brinkhof B Spee B Rothuizen J Penning LC Development and evaluation of canine reference genes for accurate quantification of gene expression Analytical Biochemistry 2006 356 36 43 10.1016/j.ab.2006.06.001 16844072

[B46] Etschmann B Wilcken B Stoevesand K Schulenburg A von der Sterner-Kock A Selection of reference genes for quantitative real-time PCR analysis in canine mammary tumors using the GeNorm algorithm Veterinary Pathology 2006 43 934 942 10.1354/vp.43-6-934 17099150

[B47] Foss DL Baarsch MJ Murtaugh MP Regulation of hypoxanthine phosphoribosyltransferase, glyceraldehyde-3-phosphate dehydrogenase and beta-actin mRNA expression in porcine immune cells and tissues Animal Biotechnology 1998 9 67 78 10.1080/10495399809525893 9676236

[B48] Robinson TL Sutherland IA Sutherland J Validation of candidate bovine reference genes for use with real-time PCR Veterinary Immunology and Immunopathology 2007 115 160 165 10.1016/j.vetimm.2006.09.012 17074403

[B49] Garcia-Crespo D Juste RA Hurtado A Selection of ovine housekeeping genes for normalisation by real-time RT-PCR; analysis of PrP gene expression and genetic susceptibility to scrapie BMC Veterinary Research 2005 1 3 10.1186/1746-6148-1-3 16188044 PMC1262732

[B50] Fitzpatrick R Casey OM Morris D Smith T Powell R Sreenan JM Postmortem stability of RNA isolated from bovine reproductive tissues Biochimica et Biophysica Acta 2002 1574 10 14 11955609 10.1016/s0167-4781(01)00322-0

[B51] Cappelli K Felicetti M Capomaccio S Spinsanti G Silvestrelli M Supplizi AV Exercise induced stress in horses: selection of the most stable reference genes for quantitative RT-PCR normalization BMC Molecular Biology 2008 9 49 10.1186/1471-2199-9-49 18489742 PMC2412902

[B52] Bogaert L Van Poucke M De Baere C Peelman L Gasthuys F Martens A Selection of a set of reliable reference genes for quantitative real-time PCR in normal equine skin and in equine sarcoids BMC Biotechnol 2006 6 24 10.1186/1472-6750-6-24 16643647 PMC1484482

[B53] Lee PD Sladek R Greenwood CM Hudson TJ Control genes and variability: absence of ubiquitous reference transcripts in diverse mammalian expression studies Genome Research 2002 12 292 297 10.1101/gr.217802 11827948 PMC155273

[B54] Dheda K Huggett JF Chang JS Kim LU Bustin SA Johnson MA Rook GA Zumla A The implications of using an inappropriate reference gene for real-time reverse transcription PCR data normalization Analytical Biochemistry 2005 344 141 143 10.1016/j.ab.2005.05.022 16054107

[B55] Fu LY Jia HL Dong QZ Wu JC Zhao Y Zhou HJ Ren N Ye QH Qin LX Suitable reference genes for real-time PCR in human HBV-related hepatocellular carcinoma with different clinical prognoses BMC Cancer 2009 9 49 10.1186/1471-2407-9-49 19200351 PMC2644316

[B56] Tatsumi K Ohashi K Taminishi S Okano T Yoshioka A Shima M Reference gene selection for real-time RT-PCR in regenerating mouse livers Biochemical and Biophysical Research Communications 2008 374 106 110 10.1016/j.bbrc.2008.06.103 18602371

[B57] Julin K Johansen LH Sommer AI Reference genes evaluated for use in infectious pancreatic necrosis virus real-time RT-qPCR assay applied during different stages of an infection Journal of Virological Methods 2009 162 30 39 10.1016/j.jviromet.2009.07.003 19638286

