# Supplementary material for: Quantitative TaqMan® real-time PCR assays for gene expression normalisation in feline tissues
Source: BMC Mol Biol. 2009 Dec 11;10:106. doi: 10.1186/1471-2199-10-106 (PMC2803789; doi:10.1186/1471-2199-10-106)
Supplement: Additional file 1 — NormFinder output. Stability values and standard errors calculated by the NormFinder program. In addition, a gene ranking for every tissue is shown. [file 1471-2199-10-106-S1.PDF]

Additional File 1

Adrenal gland

Gene name	Stability value	Standard error	Ranking
ABL	0.273	0.073	3
ACTB	0.305	0.079	5
GAPDH	0.354	0.088	6
B2M	0.296	0.077	4
GUSB	0.504	0.118	10
HMBS	0.435	0.104	8
HPRT	0.147	0.056	1
RPS7	0.243	0.068	2
YWHAZ	0.452	0.107	9
18S	0.365	0.090	7

Pancreas

Gene name	Stability value	Standard error	Ranking
ABL	1.190	0.265	7
ACTB	2.025	0.423	10
GAPDH	1.125	0.253	6
B2M	0.566	0.166	5
GUSB	0.306	0.158	2
HMBS	0.541	0.164	4
HPRT	1.520	0.326	9
RPS7	0.200	0.188	1
YWHAZ	0.384	0.154	3
18S	1.321	0.289	8

Parathyroid

Gene name	Stability value	Standard error	Ranking
ABL	0.470	0.159	6
ACTB	0.065	0.203	1
GAPDH	0.327	0.130	3
B2M	0.627	0.198	7
GUSB	0.692	0.214	8
HMBS	0.441	0.153	4
HPRT	1.062	0.314	10
RPS7	0.159	0.121	2
YWHAZ	0.469	0.159	5
18S	0.749	0.230	9

Thyroid

Gene name	Stability value	Standard error	Ranking
ABL	0.701	0.187	7
ACTB	0.213	0.127	2
GAPDH	1.230	0.300	9
B2M	0.378	0.132	5
GUSB	0.205	0.128	1
HMBS	0.358	0.130	4
HPRT	1.250	0.304	10
RPS7	0.487	0.147	6
YWHAZ	0.271	0.124	3
18S	1.215	0.297	8

Bone marrow

Gene name	Stability value	Standard error	Ranking
ABL	0.456	0.152	3
ACTB	0.649	0.180	6
GAPDH	1.512	0.349	10
B2M	0.857	0.217	7
GUSB	0.558	0.165	4
HMBS	0.388	0.146	2
HPRT	1.476	0.342	9
RPS7	0.320	0.144	1
YWHAZ	0.620	0.175	5
18S	1.322	0.310	8

Mesenteric lymph node

Gene name	Stability value	Standard error	Ranking
ABL	0.370	0.108	6
ACTB	0.222	0.087	2
GAPDH	0.484	0.129	8
B2M	0.303	0.097	3
GUSB	0.194	0.086	1
HMBS	0.326	0.100	4
HPRT	1.035	0.249	10
RPS7	0.431	0.119	7
YWHAZ	0.328	0.101	5
18S	0.852	0.208	9

Spleen

Gene name	Stability value	Standard error	Ranking
ABL	0.447	0.134	4
ACTB	0.384	0.125	3
GAPDH	0.901	0.225	9
B2M	0.453	0.135	5
GUSB	0.159	0.122	1
HMBS	0.581	0.158	6
HPRT	0.855	0.215	8
RPS7	0.255	0.112	2
YWHAZ	0.629	0.168	7
18S	1.316	0.317	10

Parotid gland

Gene name	Stability value	Standard error	Ranking
ABL	0.141	0.155	1
ACTB	0.248	0.130	2
GAPDH	0.871	0.235	8
B2M	0.535	0.164	6
GUSB	1.040	0.273	9
HMBS	0.428	0.146	5
HPRT	0.838	0.227	7
RPS7	0.261	0.130	3
YWHAZ	0.349	0.135	4
18S	1.518	0.386	10

Additional File 1

**Duodenum**				**Ileum**			
Gene name	Stability value	Standard error	Ranking	Gene name	Stability value	Standard error	Ranking
ABL	0.775	0.218	6	ABL	0.232	0.119	2
ACTB	0.513	0.176	3	ACTB	0.351	0.125	4
GAPDH	0.413	0.167	2	GAPDH	0.597	0.164	5
B2M	1.584	0.387	9	B2M	1.015	0.251	9
GUSB	1.143	0.291	8	GUSB	0.614	0.168	6
HMBS	0.609	0.190	4	HMBS	0.093	0.186	1
HPRT	1.790	0.433	10	HPRT	1.002	0.248	8
RPS7	0.074	0.415	1	RPS7	0.827	0.211	7
YWHAZ	0.727	0.209	5	YWHAZ	0.331	0.123	3
18S	1.022	0.266	7	18S	1.192	0.290	10
**Liver**				**Kidney**			
Gene name	Stability value	Standard error	Ranking	Gene name	Stability value	Standard error	Ranking
ABL	0.453	0.131	7	ABL	0.279	0.088	3
ACTB	0.572	0.156	8	ACTB	0.310	0.091	4
GAPDH	0.283	0.101	2	GAPDH	0.537	0.122	7
B2M	0.682	0.181	9	B2M	0.715	0.152	8
GUSB	0.282	0.100	1	GUSB	0.452	0.109	6
HMBS	0.337	0.109	5	HMBS	0.368	0.097	5
HPRT	0.315	0.105	4	HPRT	0.830	0.172	9
RPS7	0.289	0.102	3	RPS7	0.222	0.085	2
YWHAZ	0.402	0.121	6	YWHAZ	0.081	0.129	1
18S	1.110	0.282	10	18S	1.391	0.276	10
**Myocardium**				**Brain**			
Gene name	Stability value	Standard error	Ranking	Gene name	Stability value	Standard error	Ranking
ABL	0.393	0.111	6	ABL	0.239	0.069	2
ACTB	0.105	0.094	1	ACTB	0.151	0.062	1
GAPDH	0.193	0.083	3	GAPDH	0.313	0.079	5
B2M	1.108	0.265	10	B2M	0.511	0.113	8
GUSB	0.435	0.118	7	GUSB	0.347	0.084	7
HMBS	0.225	0.085	4	HMBS	0.298	0.077	4
HPRT	0.757	0.186	9	HPRT	0.334	0.082	6
RPS7	0.109	0.092	2	RPS7	0.249	0.070	3
YWHAZ	0.265	0.090	5	YWHAZ	0.249	0.070	3
18S	0.533	0.138	8	18S	1.052	0.217	9
**Blood**				**Neoplastic tissues**			
Gene name	Stability value	Standard error	Ranking	Gene name	Stability value	Standard error	Ranking
ABL	0.354	0.127	2	ABL	0.625	0.145	6
ACTB	0.524	0.156	5	ACTB	0.324	0.094	2
GAPDH	0.368	0.129	3	GAPDH	0.467	0.116	4
B2M	0.390	0.132	4	B2M	0.504	0.122	5
GUSB	0.577	0.167	6	GUSB	0.632	0.146	8
HMBS	1.174	0.302	10	HMBS	0.381	0.102	3
HPRT	0.877	0.233	8	HPRT	0.785	0.176	10
RPS7	0.887	0.235	9	RPS7	0.318	0.093	1
YWHAZ	0.350	0.126	1	YWHAZ	0.696	0.158	9
18S	0.662	0.185	7	18S	0.626	0.145	7

Additional File 1

**Lymphatic tissues**				**Endocrine tissues**			
Gene name	Stability value	Standard error	Ranking	Gene name	Stability value	Standard error	Ranking
ABL	0.991	0.144	5	ABL	0.802	0.101	6
ACTB	0.636	0.108	3	ACTB	1.227	0.143	9
GAPDH	1.036	0.149	6	GAPDH	1.028	0.123	8
B2M	1.078	0.153	7	B2M	0.464	0.072	3
GUSB	0.301	0.096	2	GUSB	0.462	0.072	2
HMBS	1.867	0.247	10	HMBS	0.518	0.076	5
HPRT	1.204	0.168	8	HPRT	1.284	0.149	10
RPS7	0.278	0.098	1	RPS7	0.509	0.075	4
YWHAZ	0.707	0.115	4	YWHAZ	0.450	0.072	1
18S	1.273	0.176	9	18S	1.023	0.123	7
**Gastrointestinal tissues**				**All tissues**			
Gene name	Stability value	Standard error	Ranking	Gene name	Stability value	Standard error	Ranking
ABL	0.626	0.103	5	ABL	0.816	0.055	4
ACTB	0.490	0.091	1	ACTB	0.881	0.058	5
GAPDH	0.700	0.111	6	GAPDH	1.186	0.074	9
B2M	1.099	0.157	8	B2M	1.019	0.065	6
GUSB	1.008	0.146	7	GUSB	0.693	0.050	2
HMBS	0.546	0.095	2	HMBS	1.025	0.066	7
HPRT	1.387	0.192	10	HPRT	1.208	0.075	10
RPS7	0.588	0.099	4	RPS7	0.449	0.041	1
YWHAZ	0.547	0.095	3	YWHAZ	0.804	0.055	3
18S	1.199	0.169	9	18S	1.143	0.072	8
