# Supplementary material for: Quantitative TaqMan® real-time PCR assays for gene expression normalisation in feline tissues
Source: BMC Mol Biol. 2009 Dec 11;10:106. doi: 10.1186/1471-2199-10-106 (PMC2803789; doi:10.1186/1471-2199-10-106)

Additional File 2
a) geNorm output: Adrenal gland

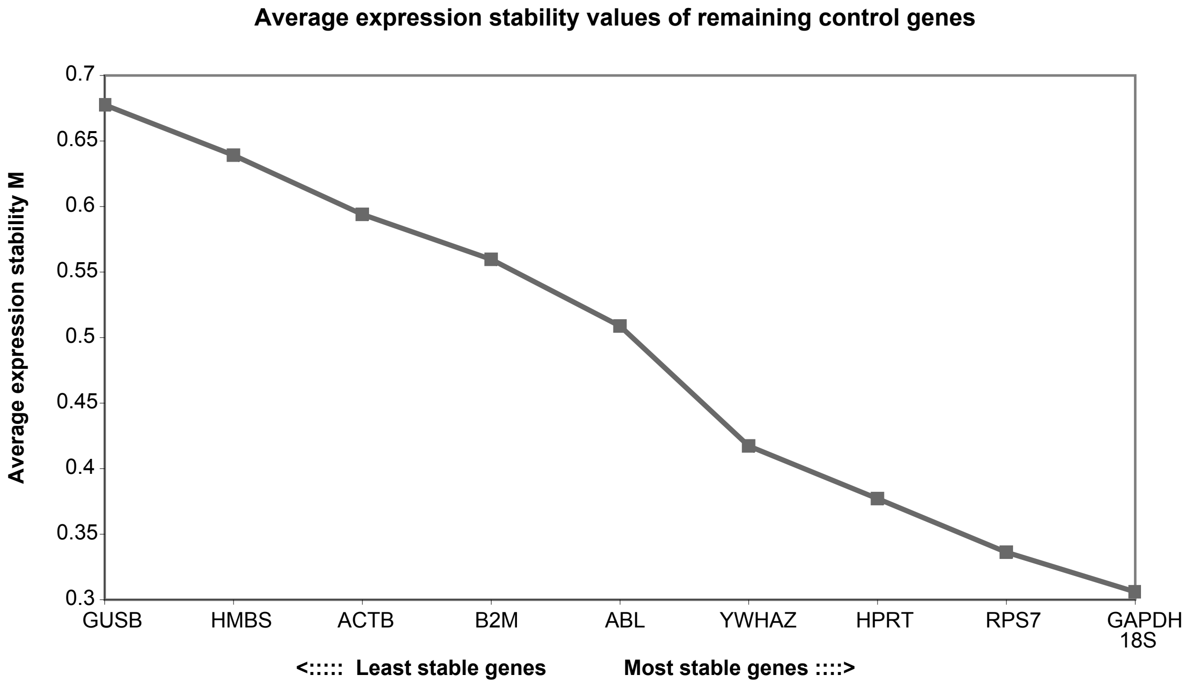

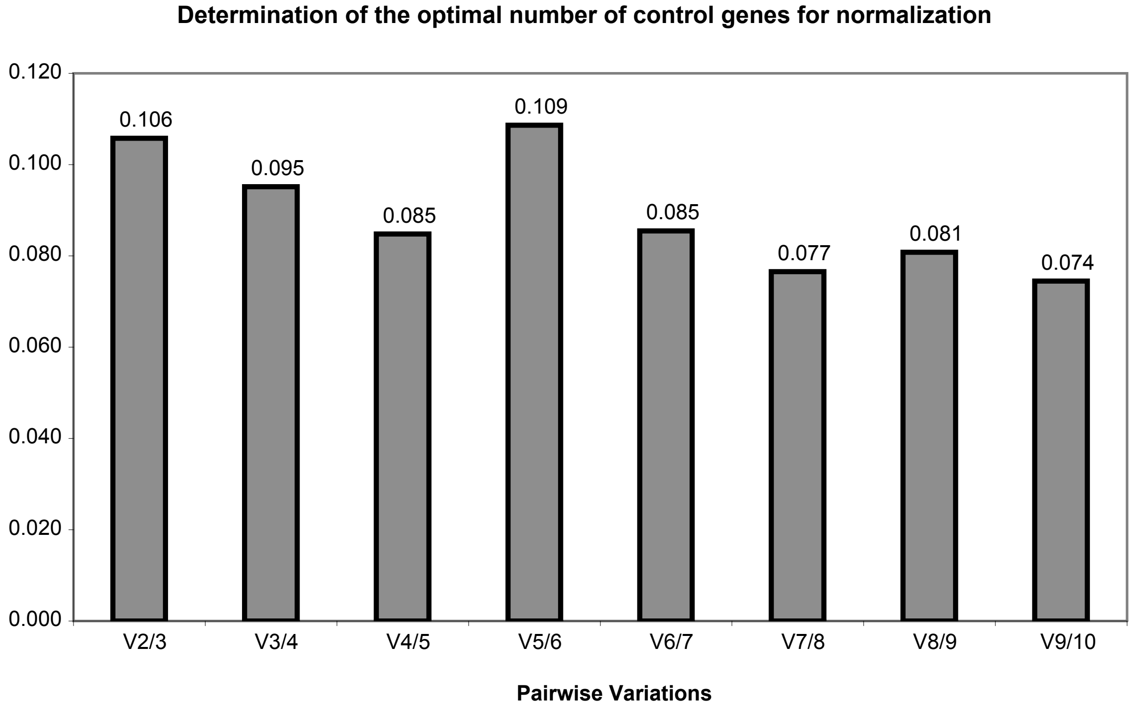

Additional File 2
b) geNorm output: Pancreas

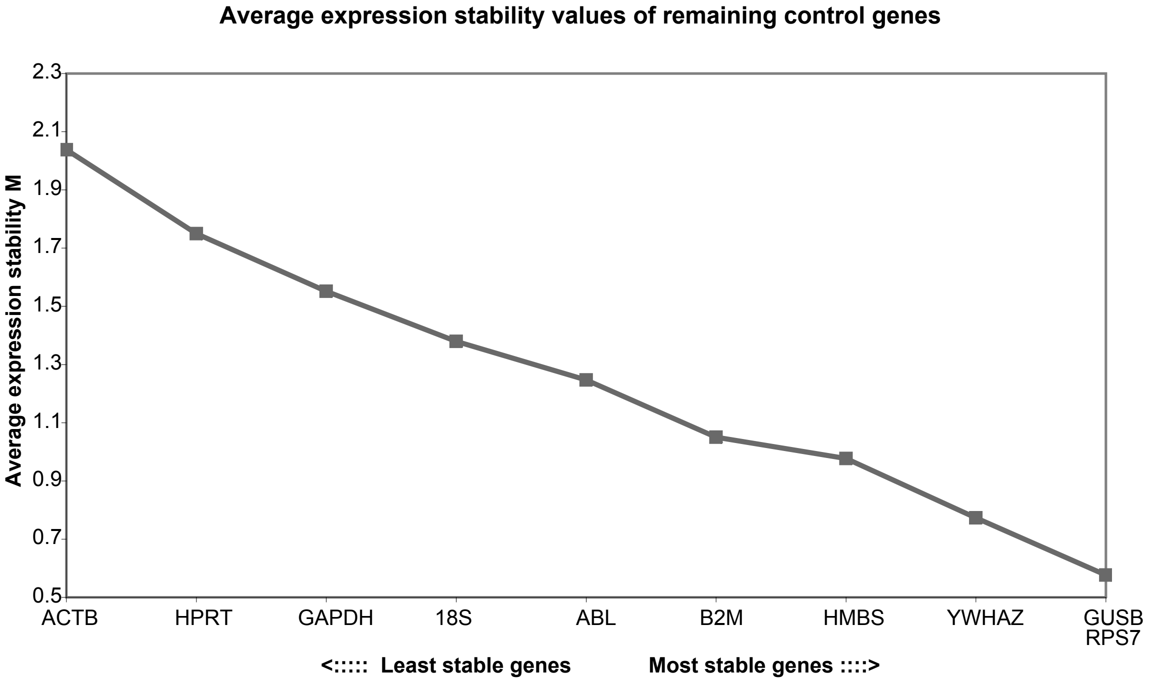

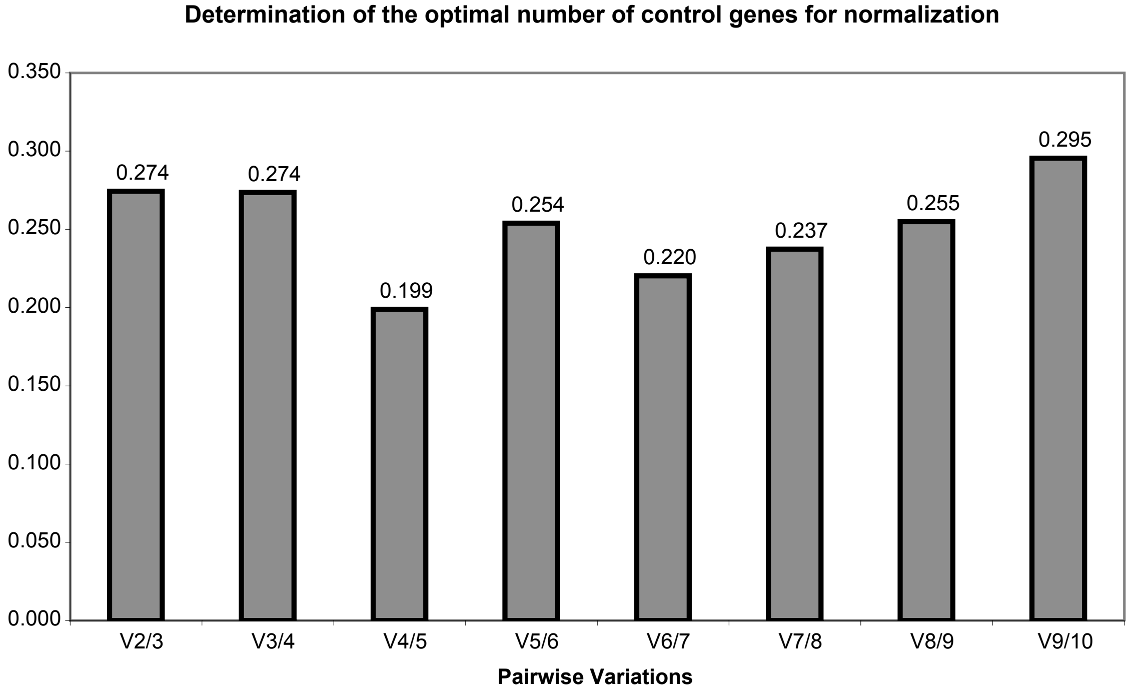

Additional File 2
c) geNorm output: Parathyroid

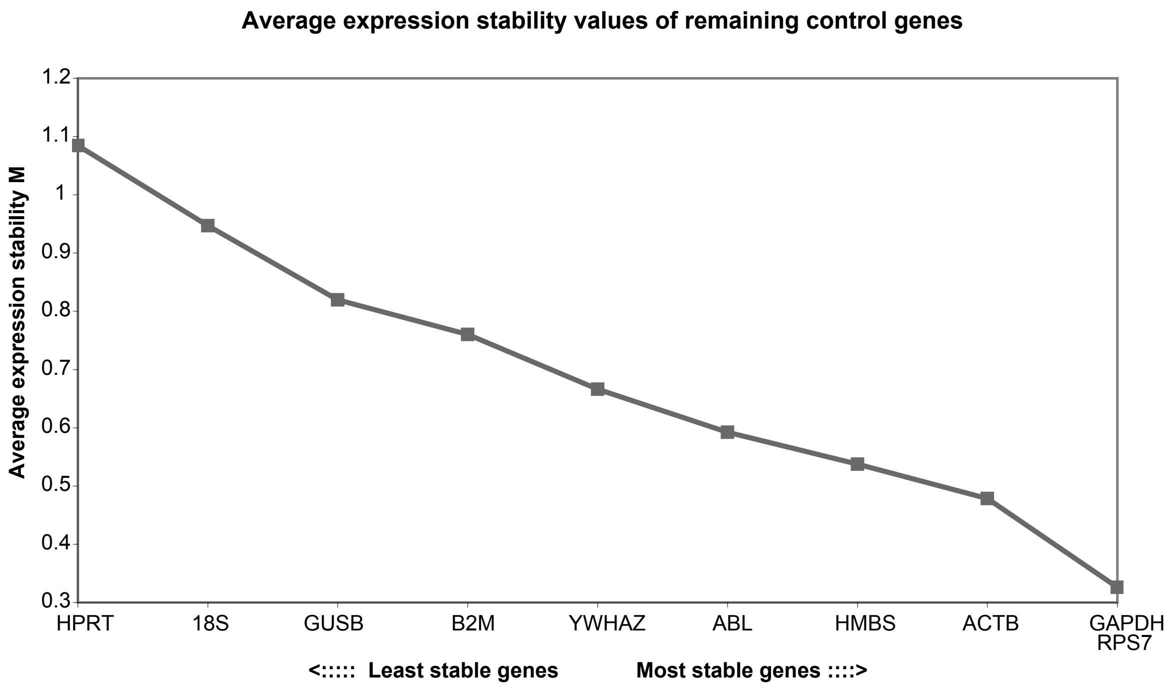

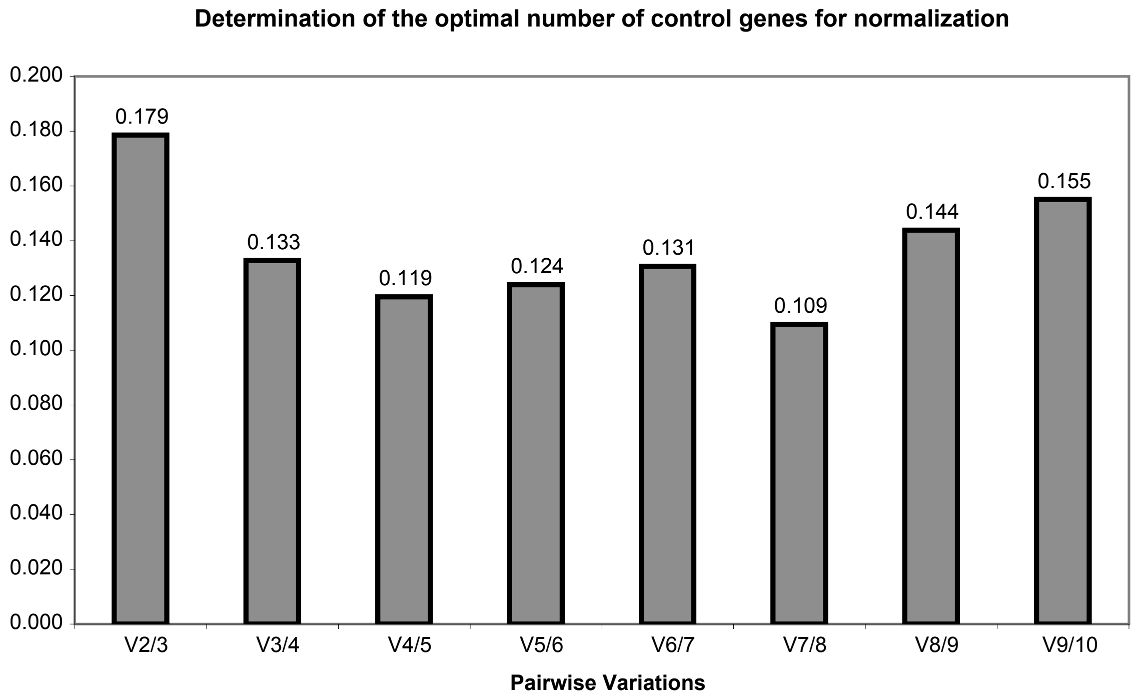

Additional File 2
d) geNorm output: Thyroid

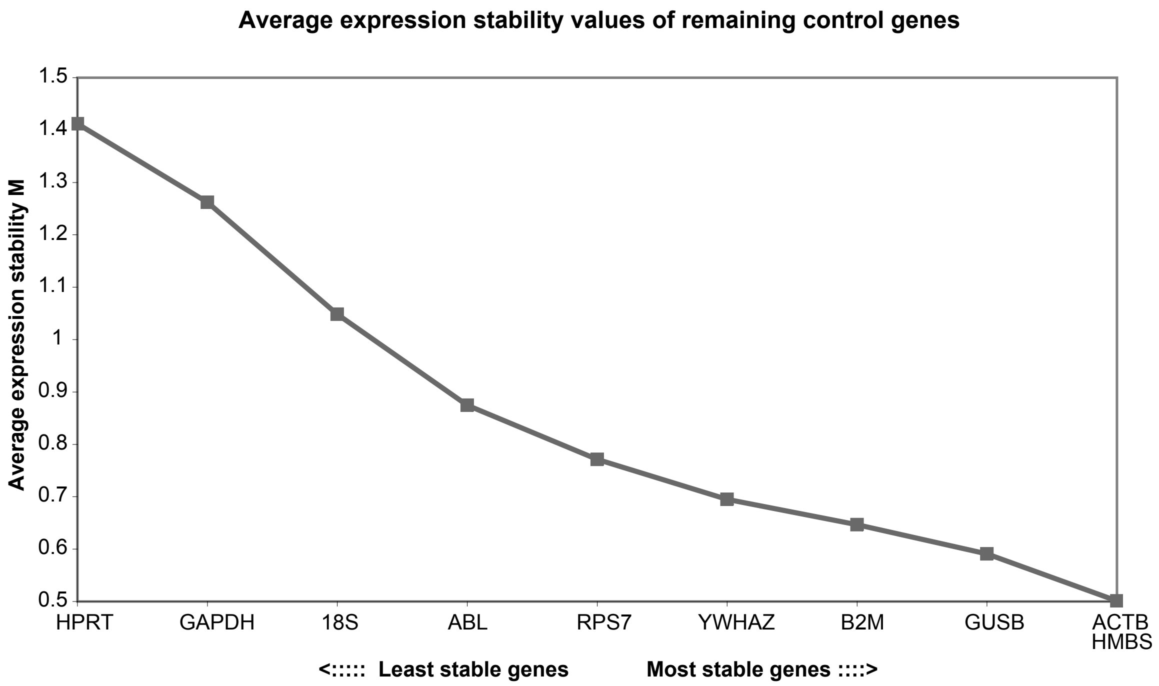

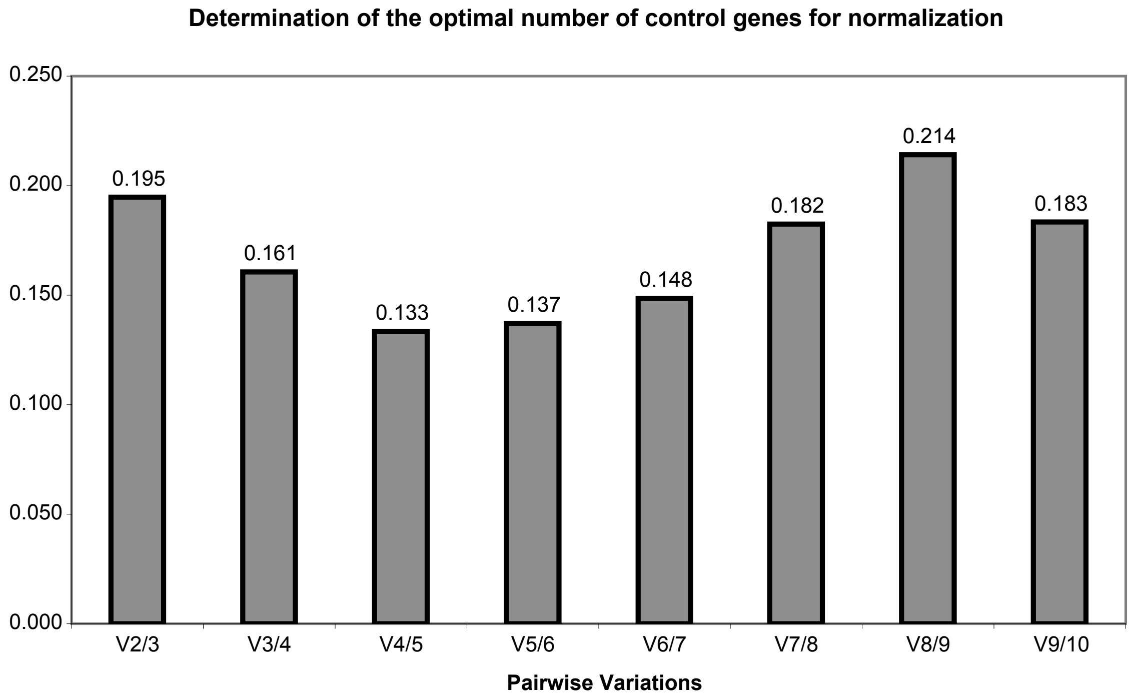

Additional File 2
e) geNorm output: Bone marrow

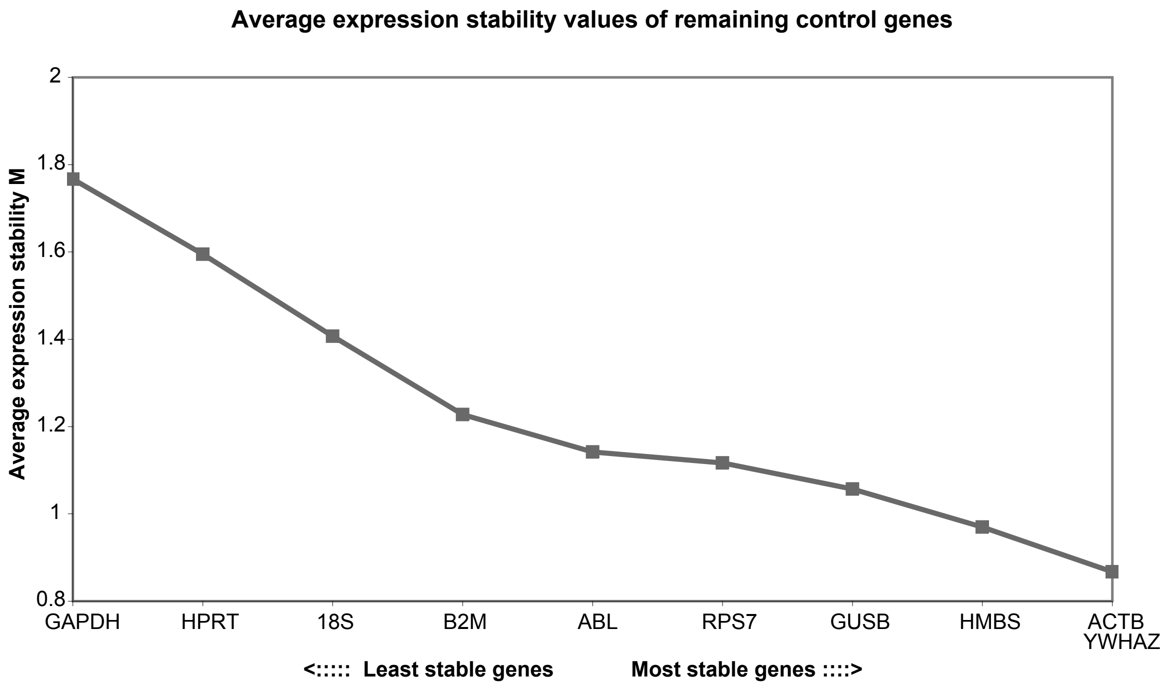

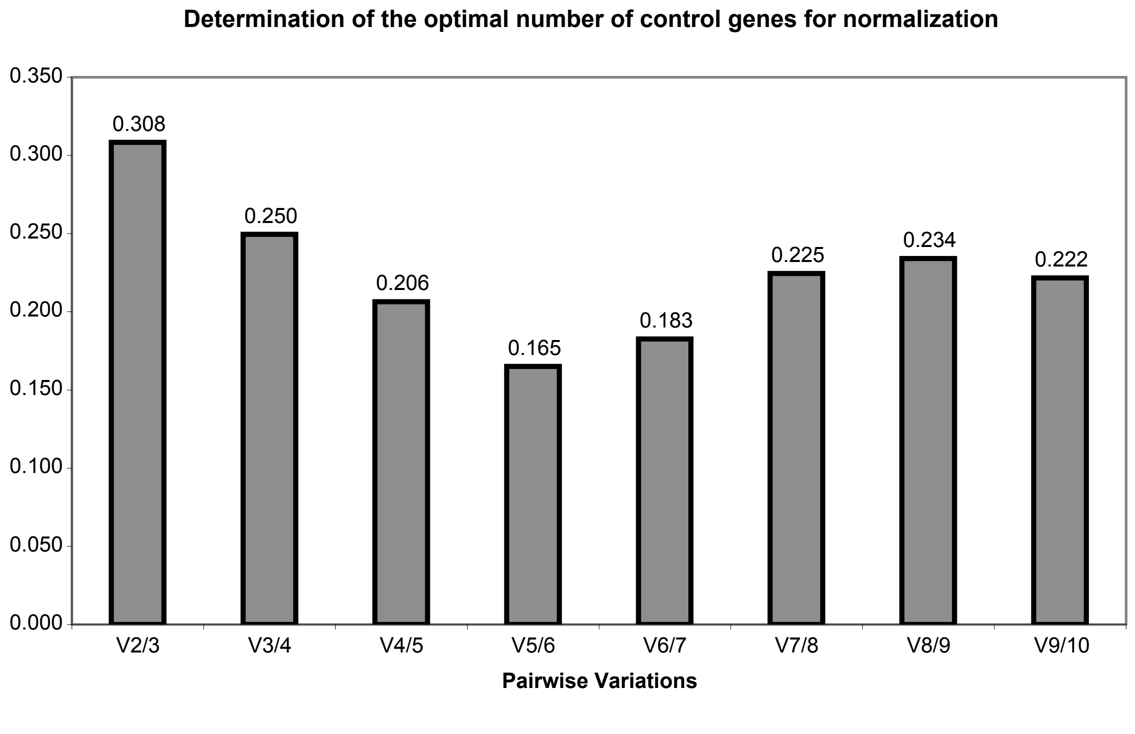

Additional File 2
f) geNorm output: Mesenteric lymph node

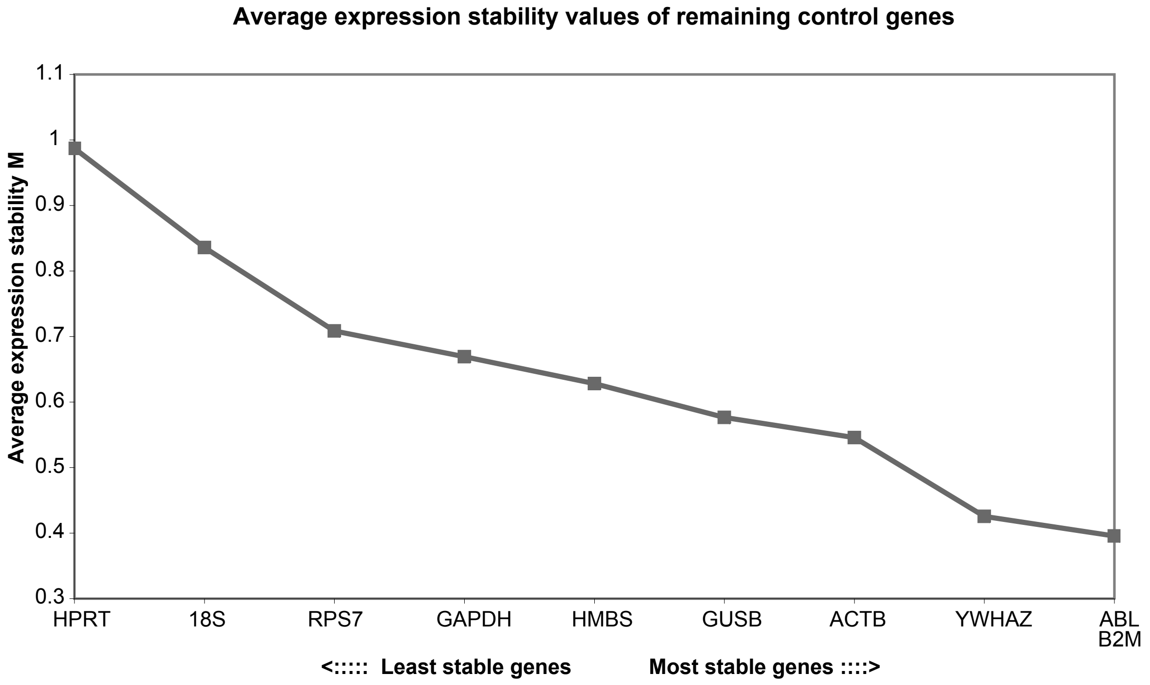

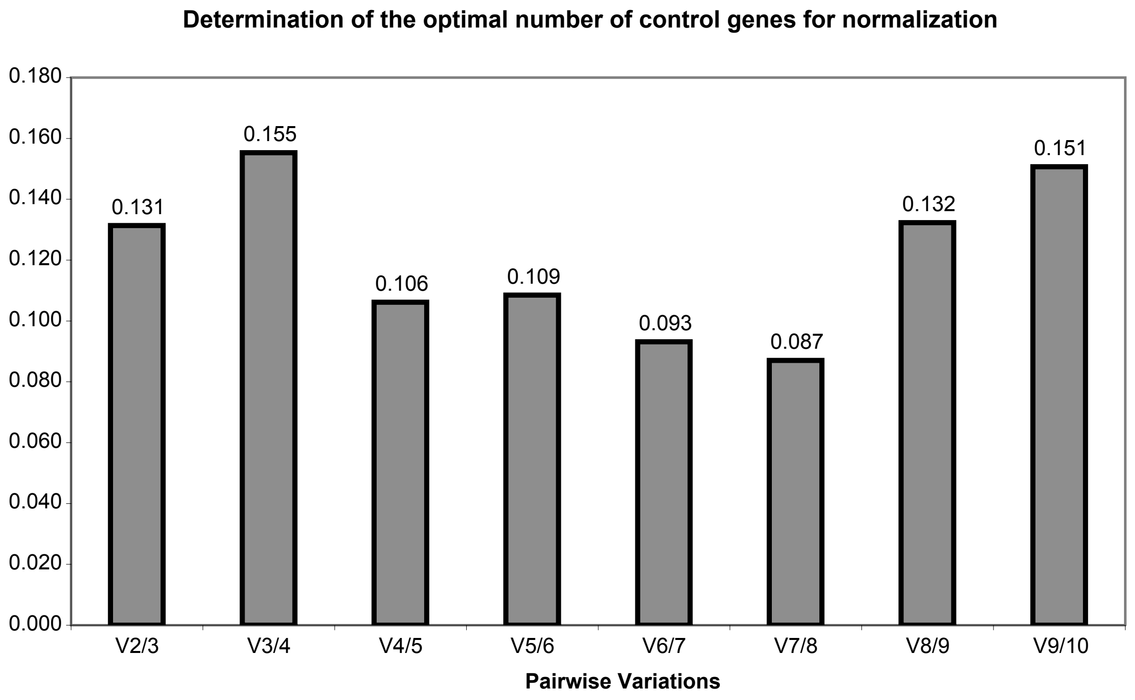

Additional File 2
g) geNorm output: Spleen

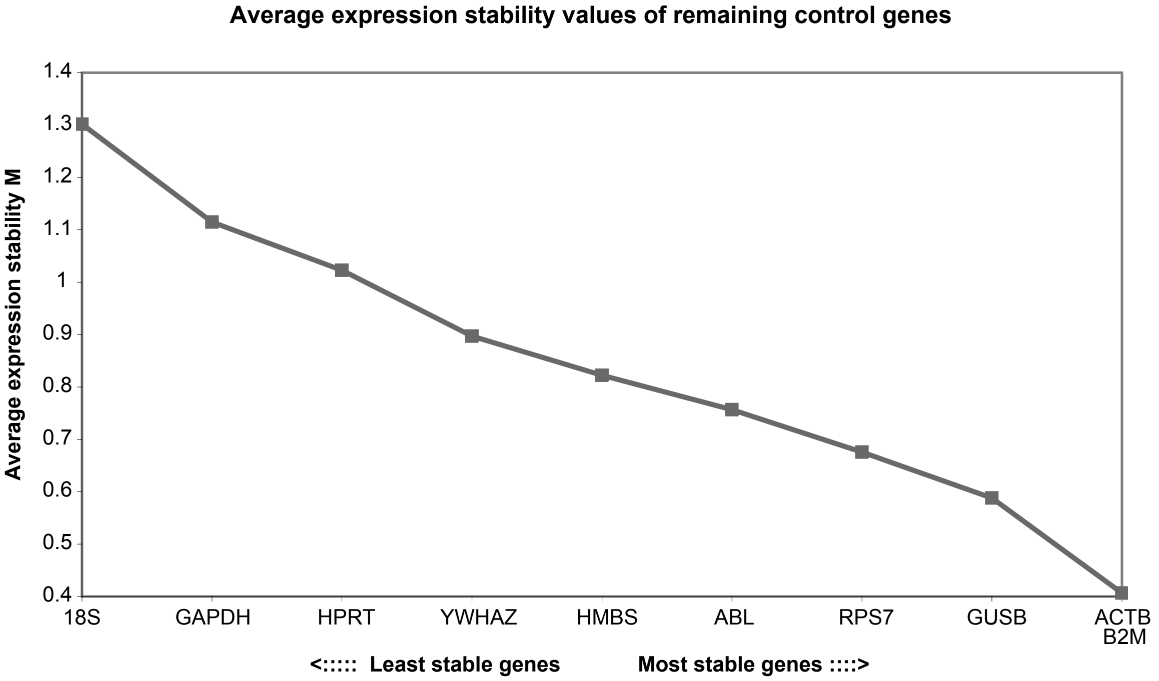

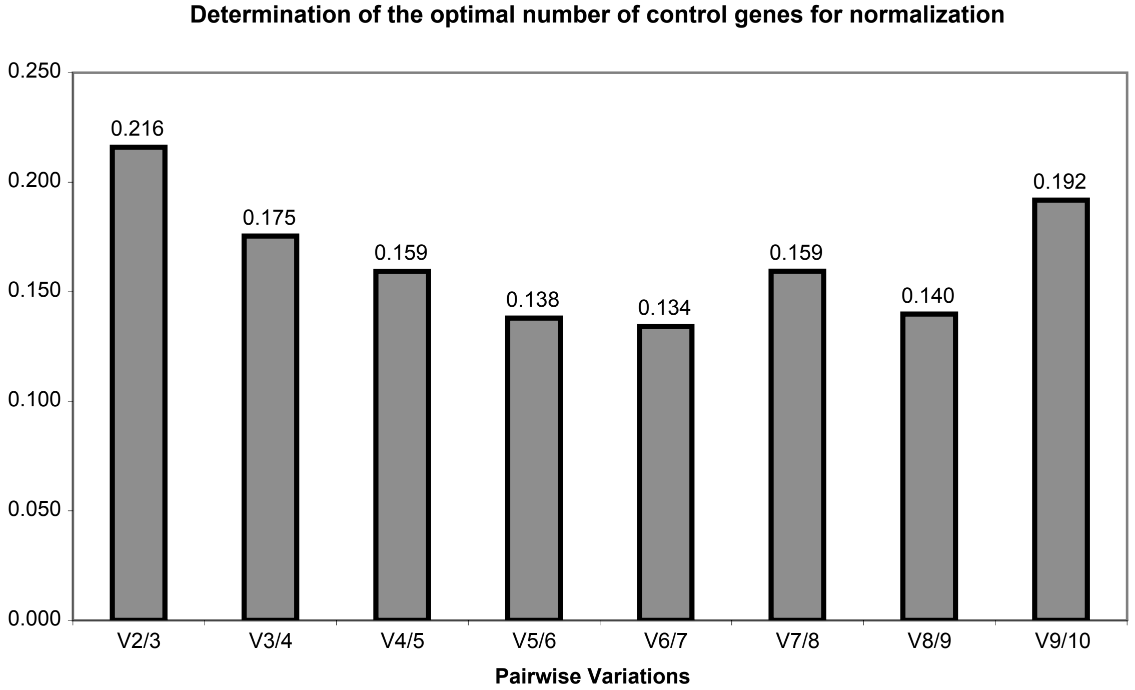

Additional File 2
h) geNorm output: Parotid gland

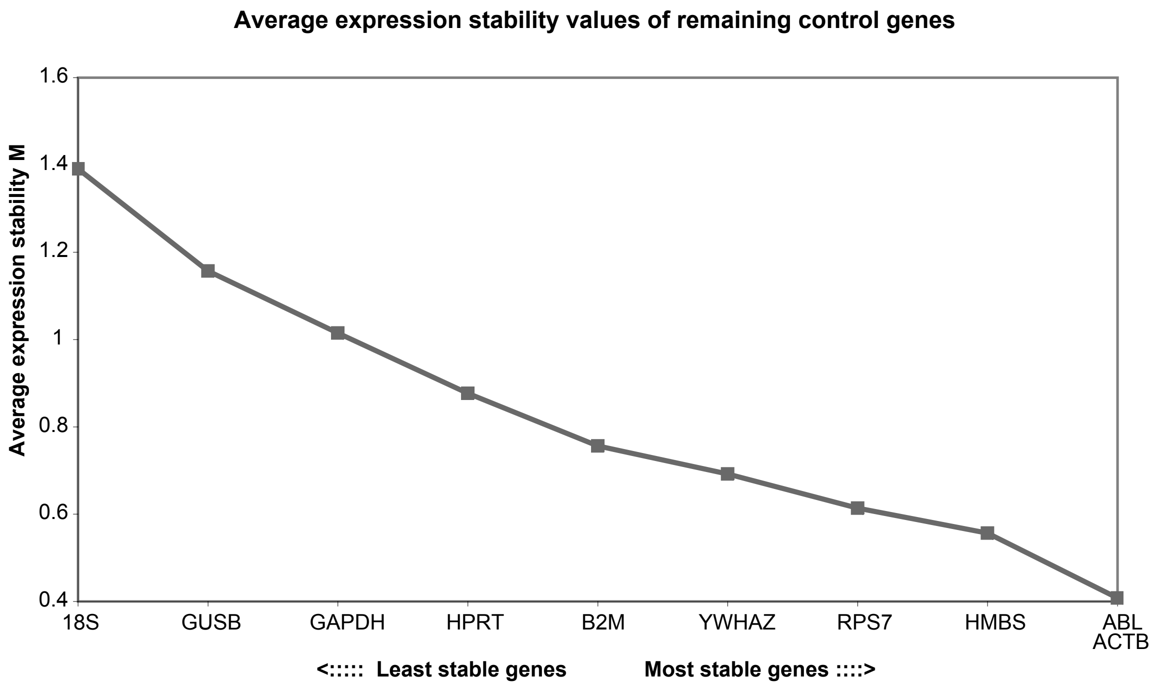

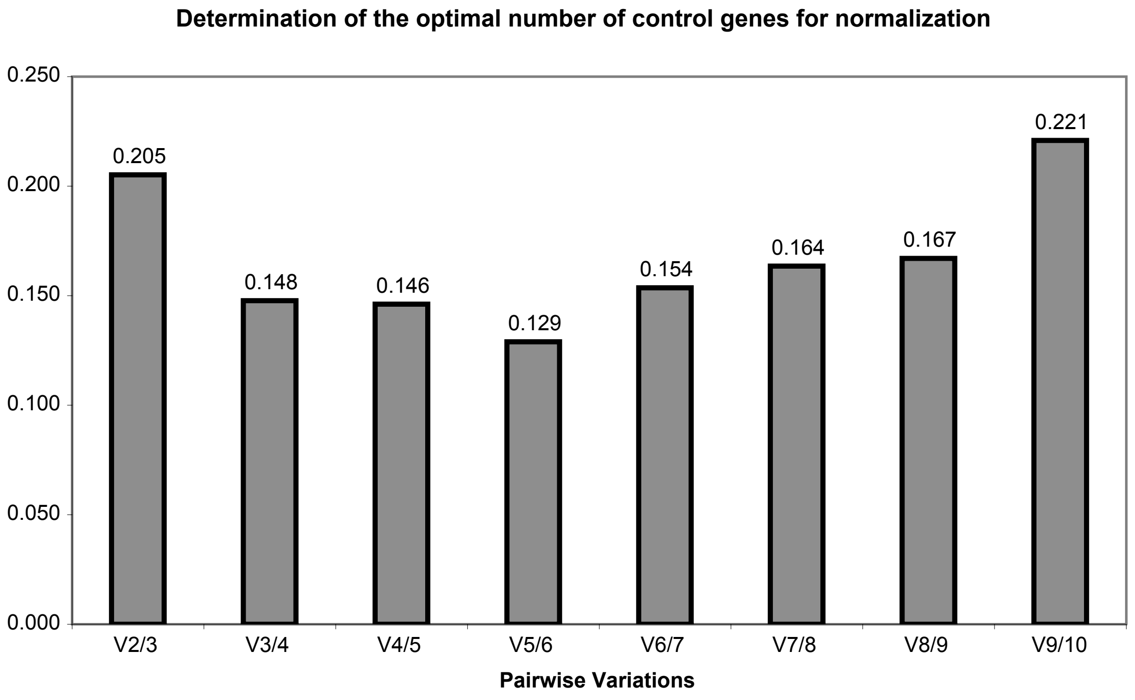

Additional File 2
i) geNorm output: Duodenum

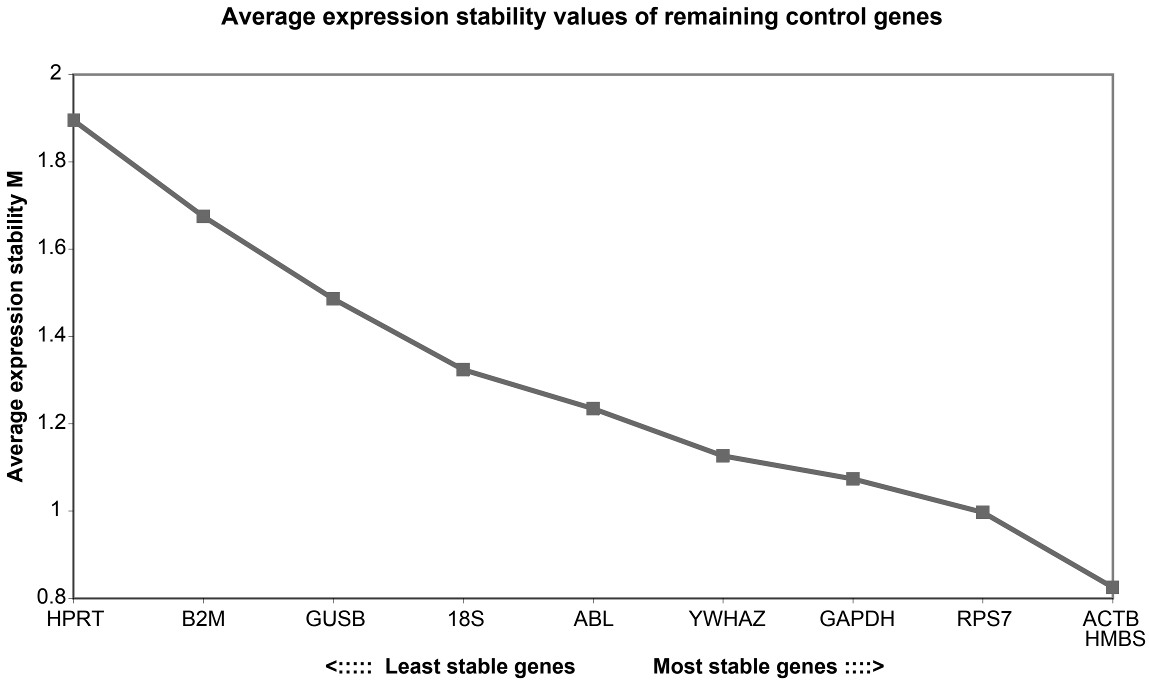

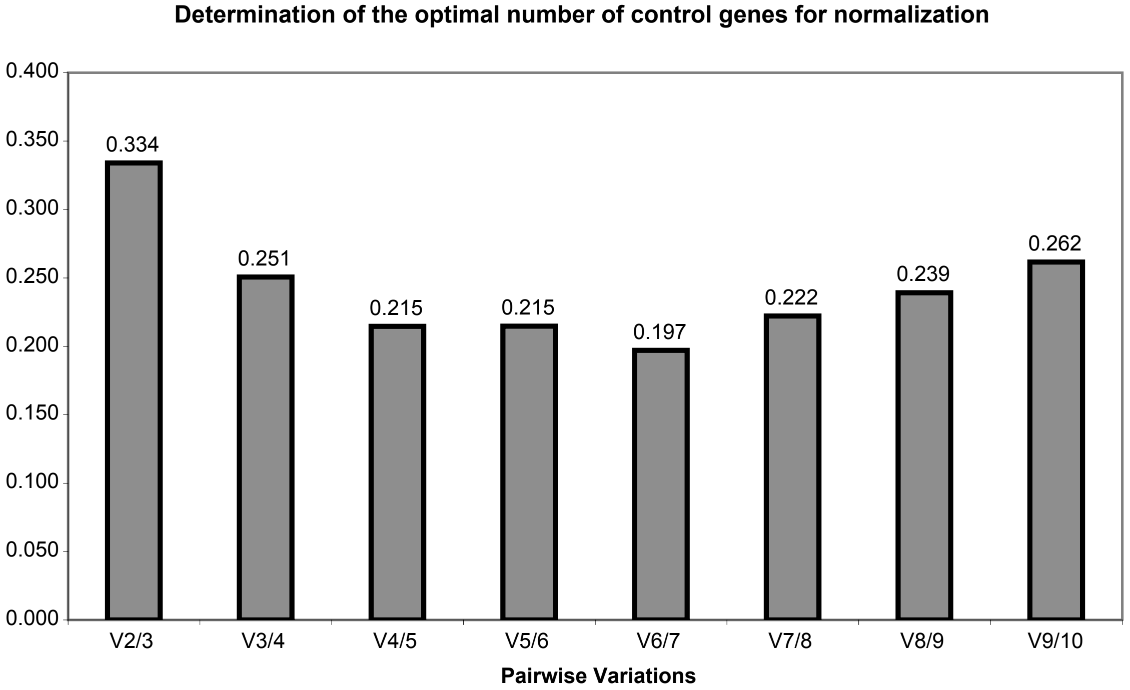

Additional File 2
j) geNorm output: Ileum

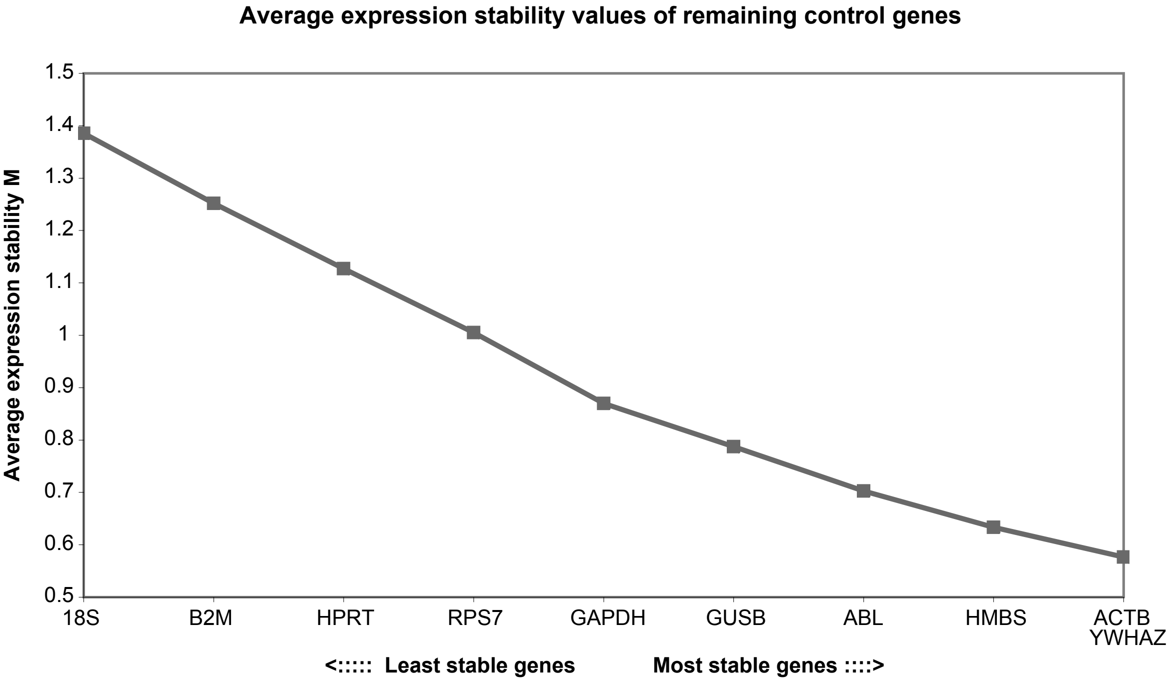

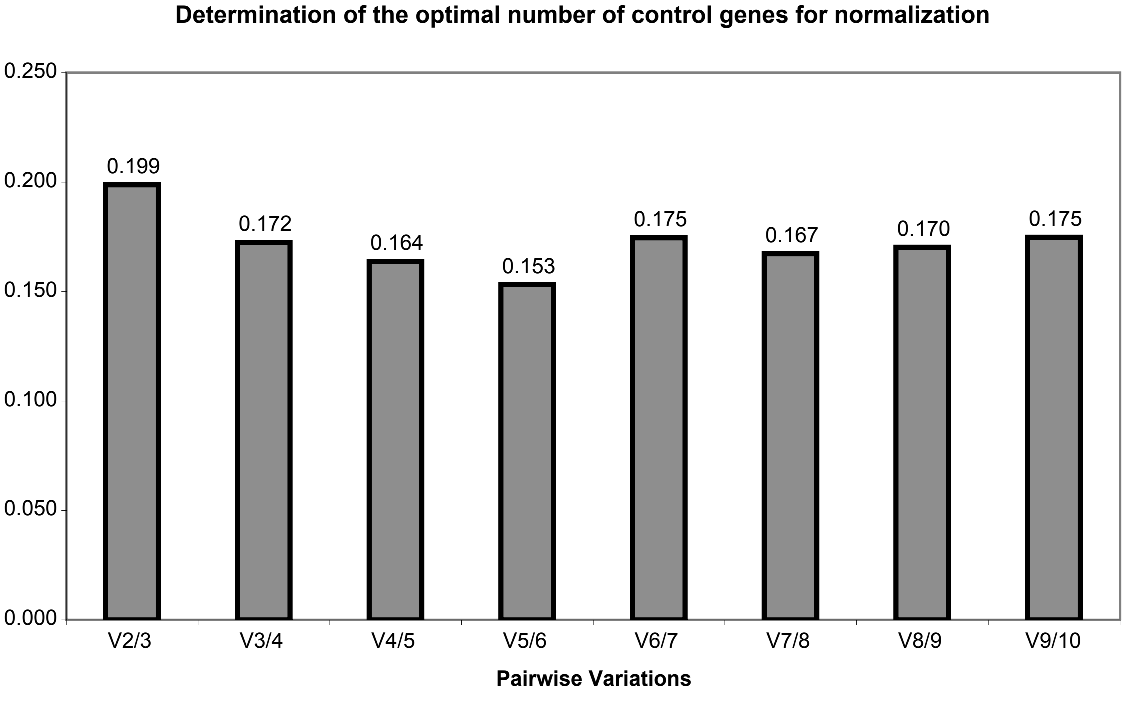

Additional File 2
k) geNorm output: Liver

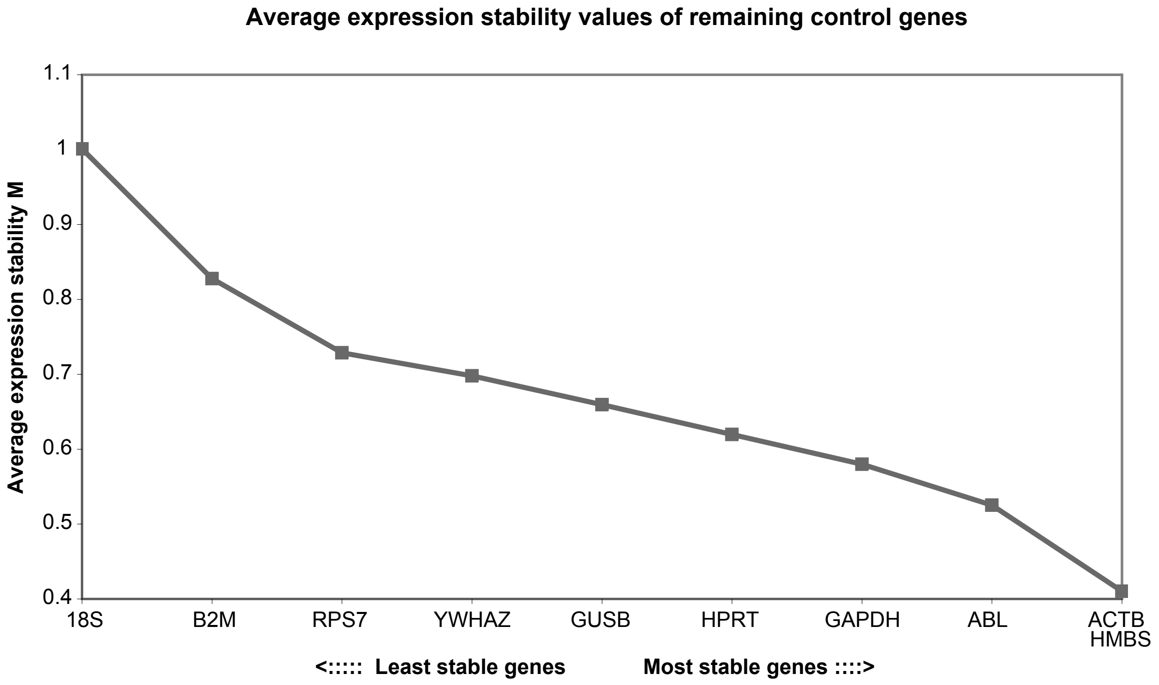

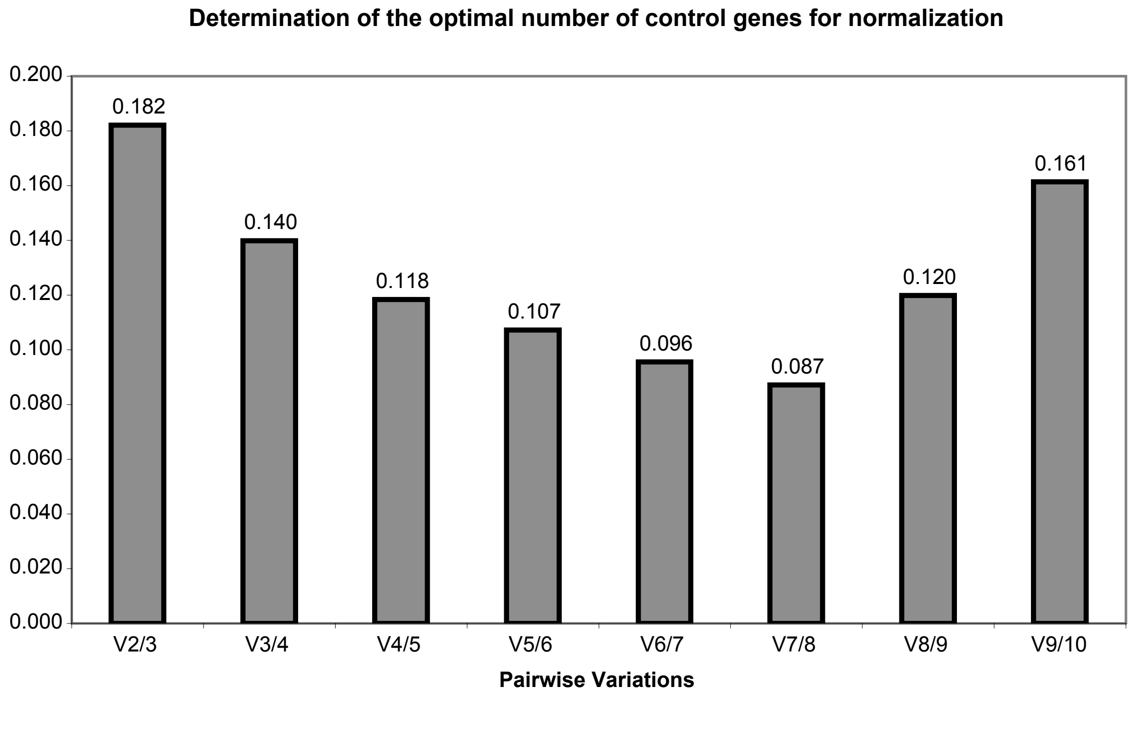

Additional File 2
I) geNorm output: Kidney

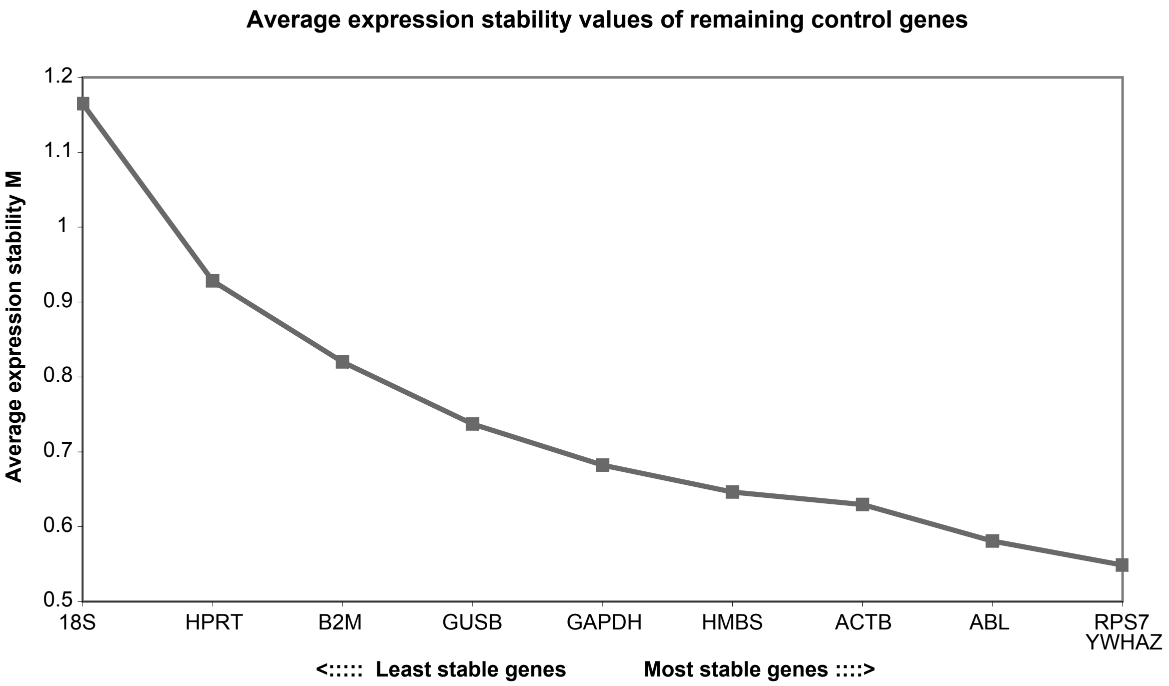

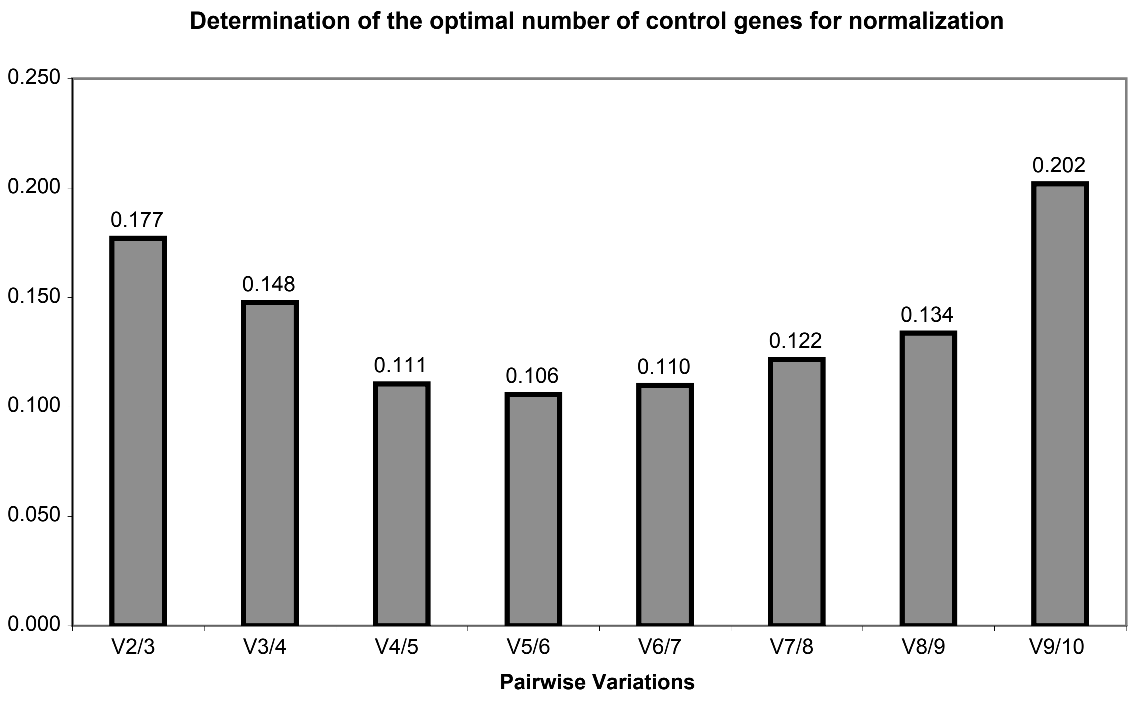

Additional File 2
m) geNorm output: Myocardium

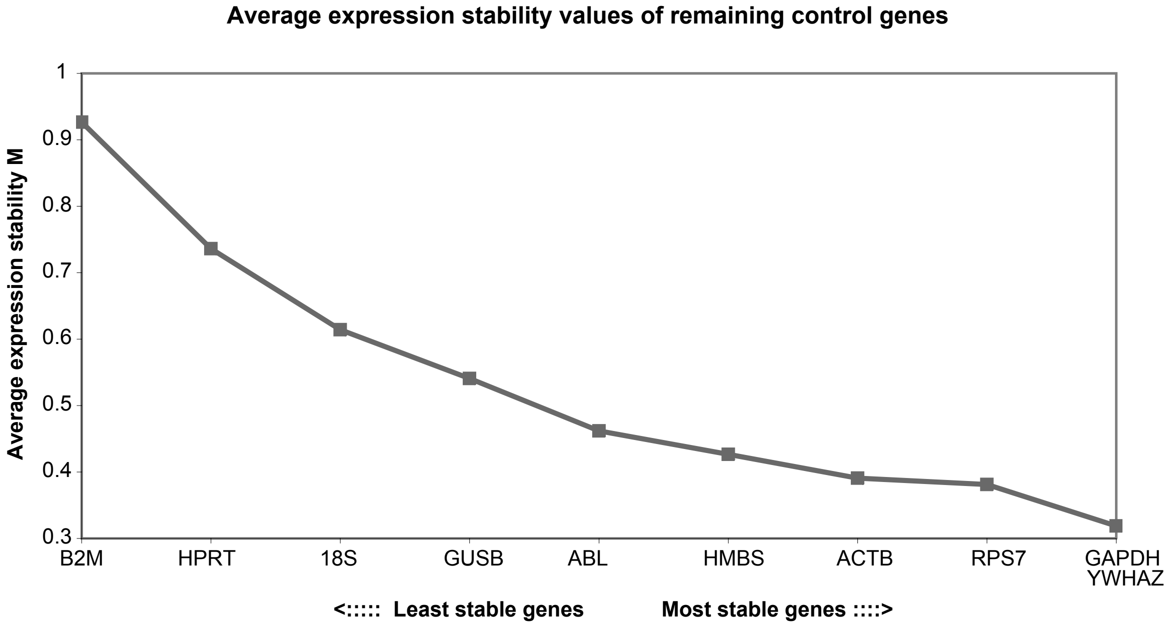

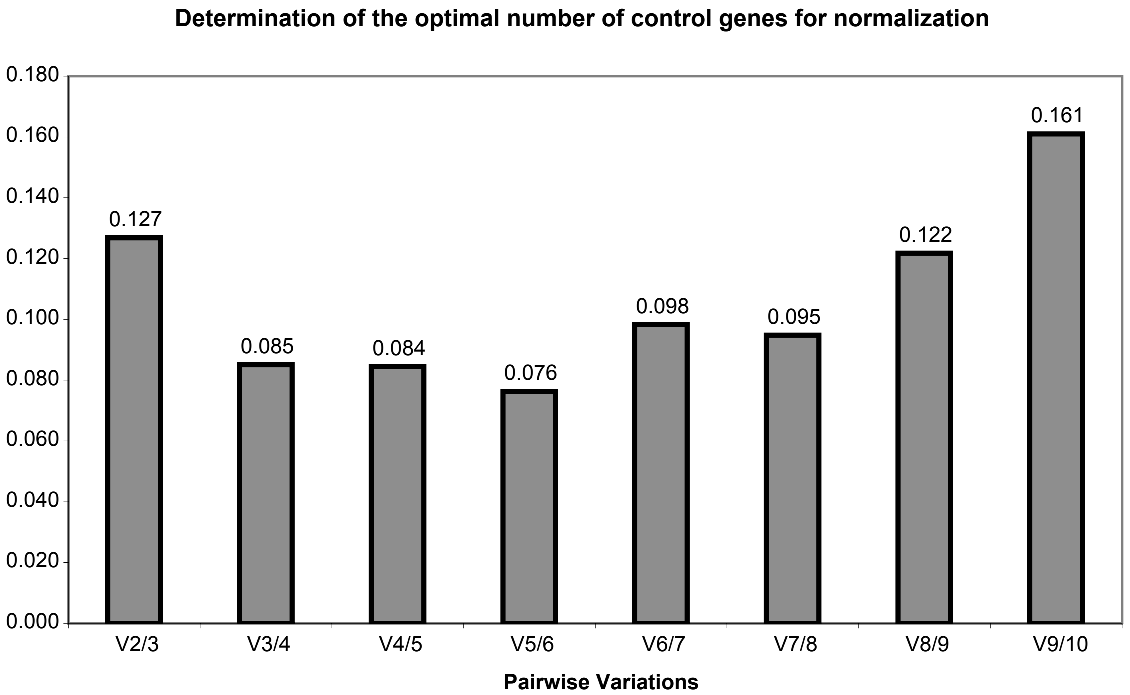

Additional File 2
n) geNorm output: Brain

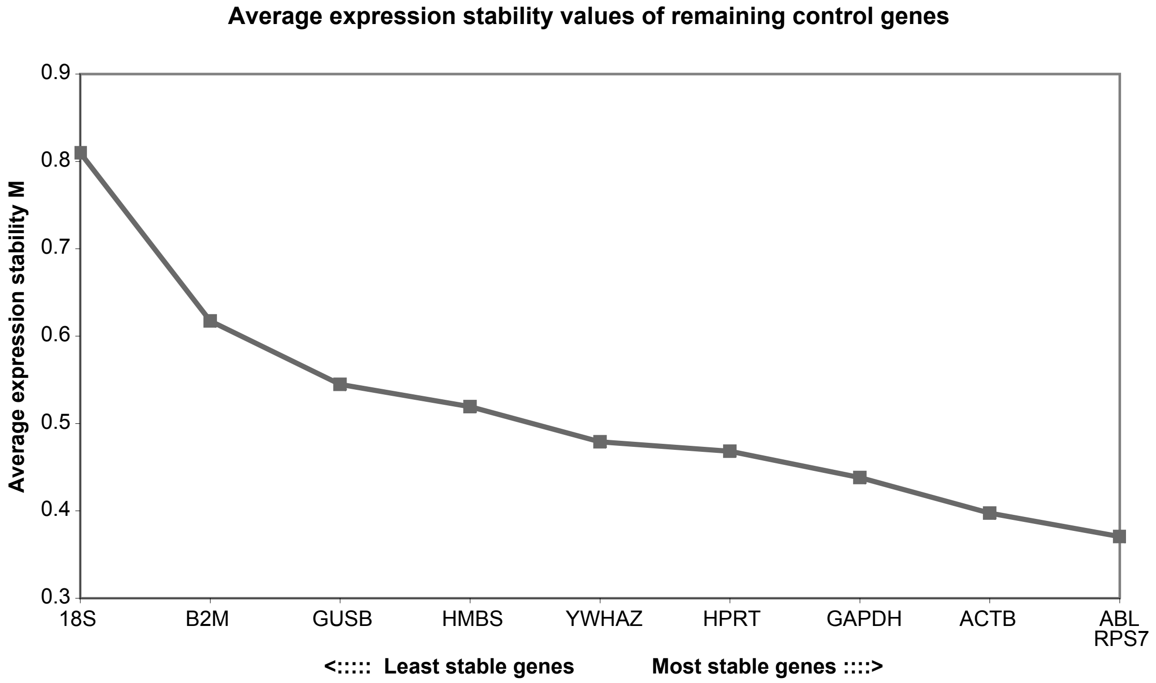

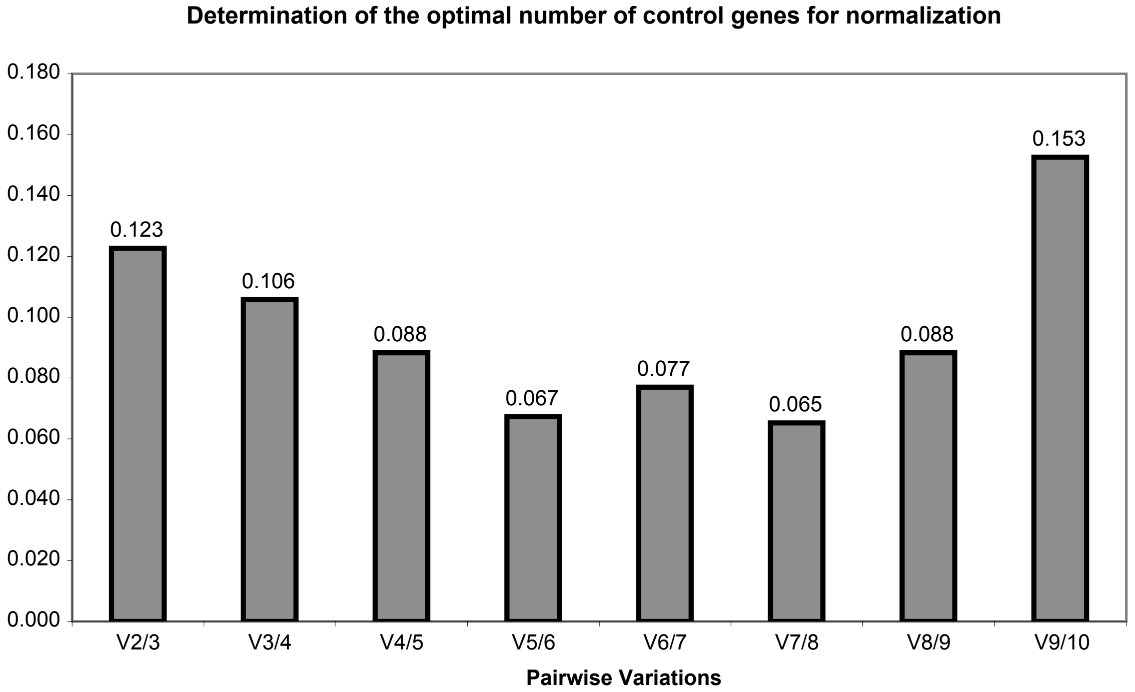

Additional File 2
o) geNorm output: Blood

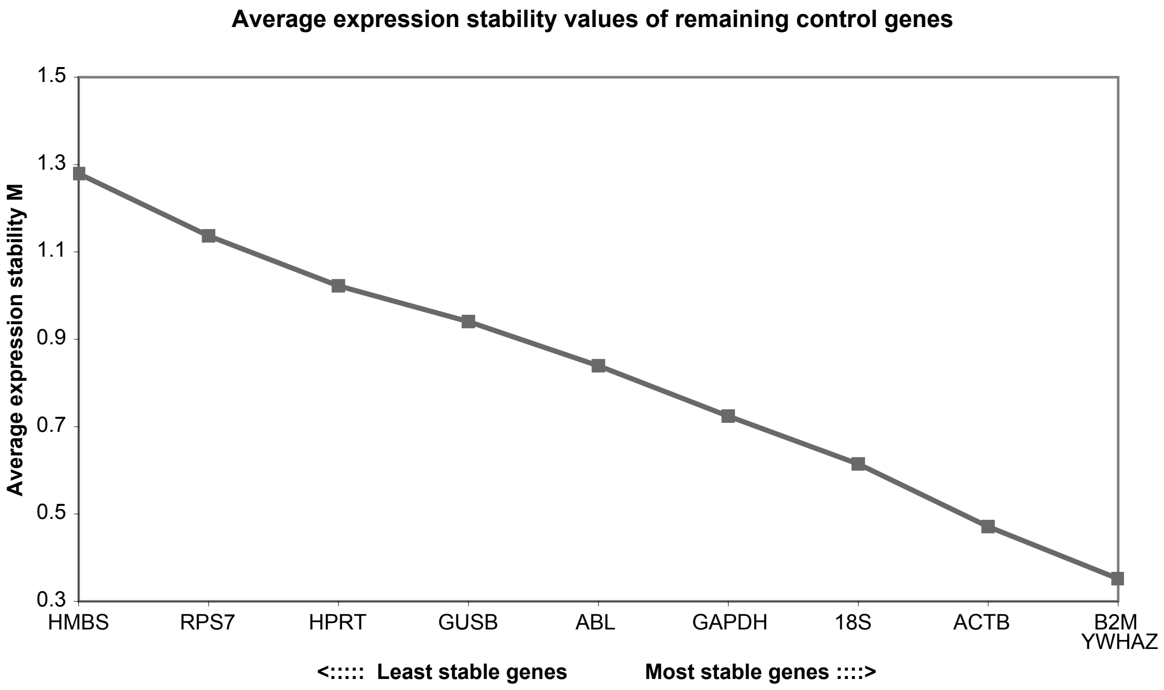

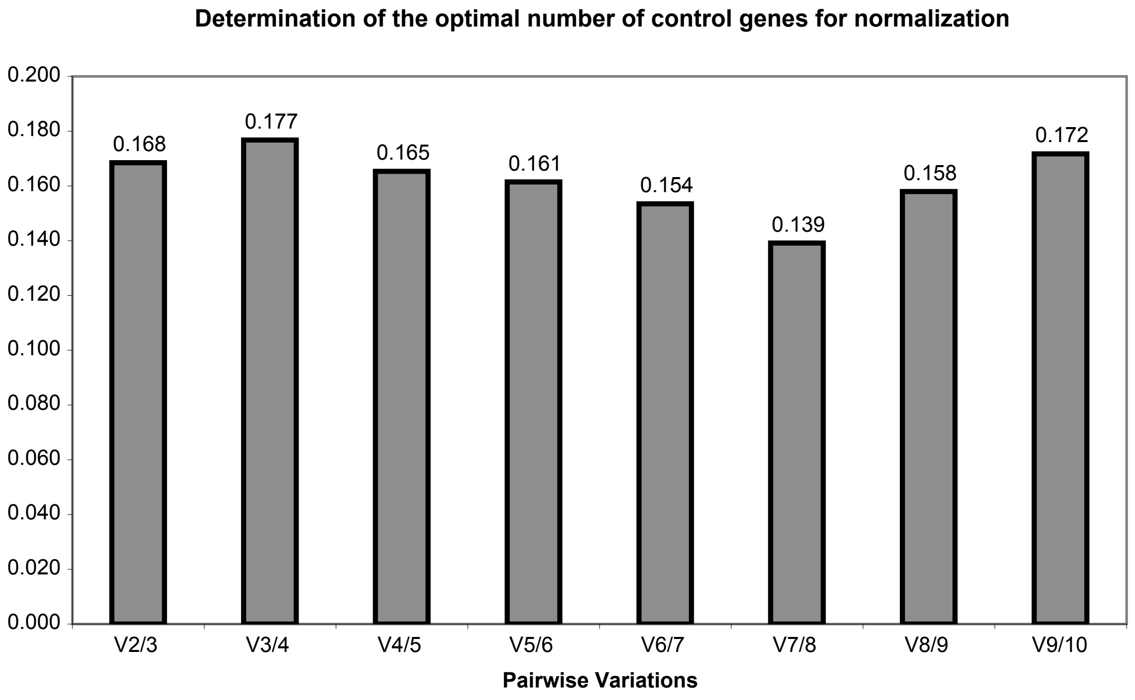

Additional File 2
p) geNorm output: Neoplastic tissues

Average expression stability values of remaining control genes

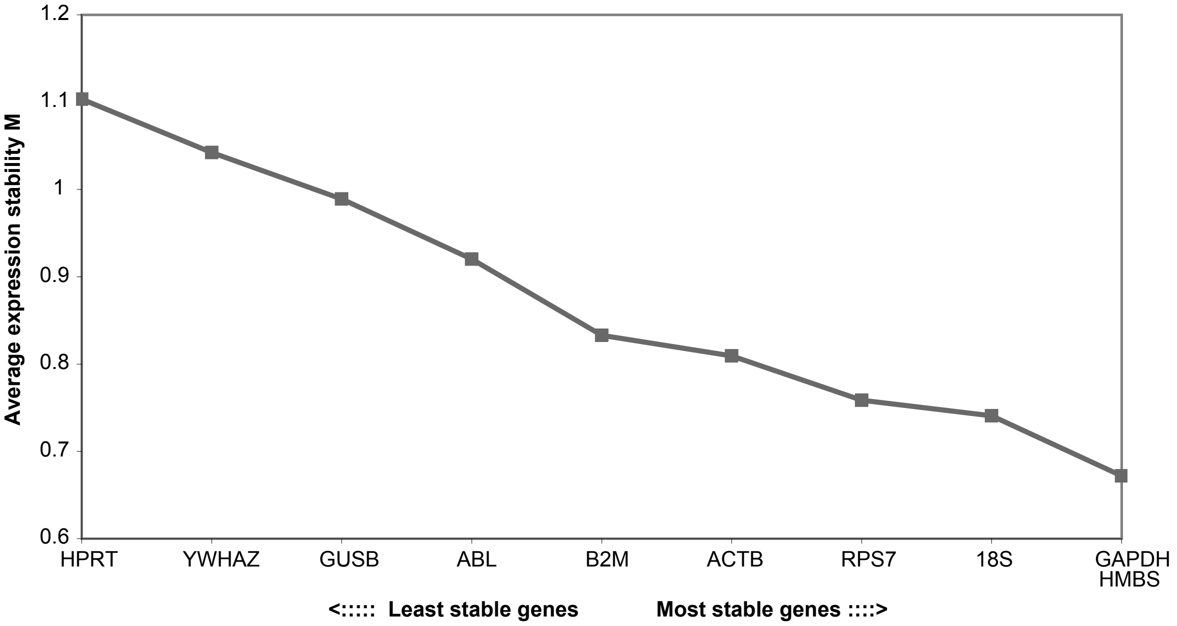

Determination of the optimal number of control genes for normalization

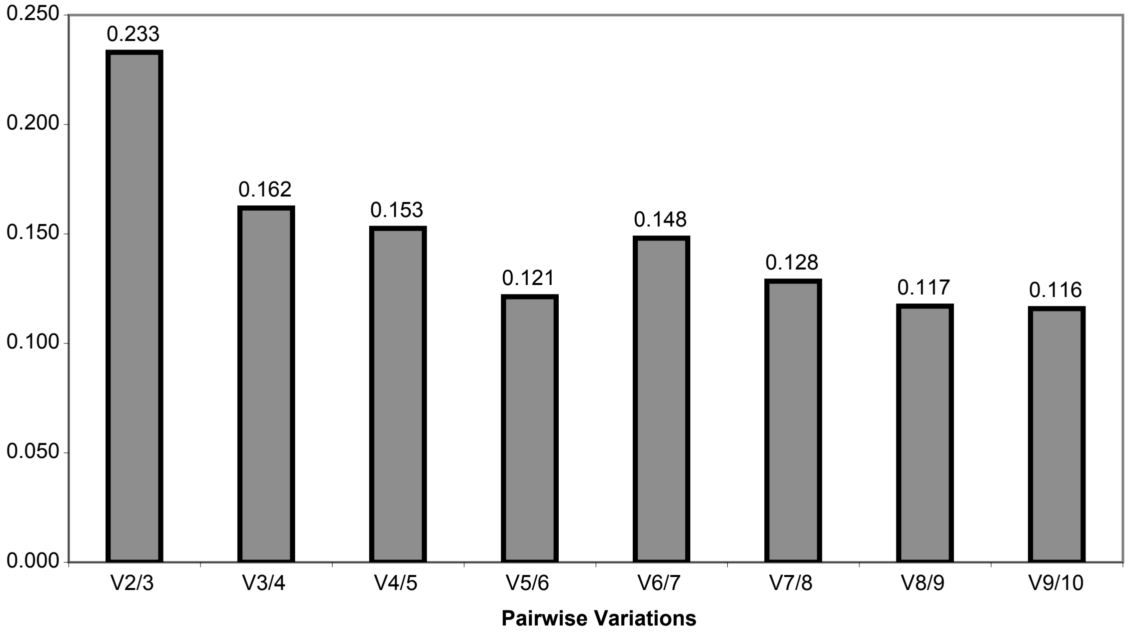

Additional File 2
q) geNorm output: All healthy tissues

Average expression stability values of remaining control genes

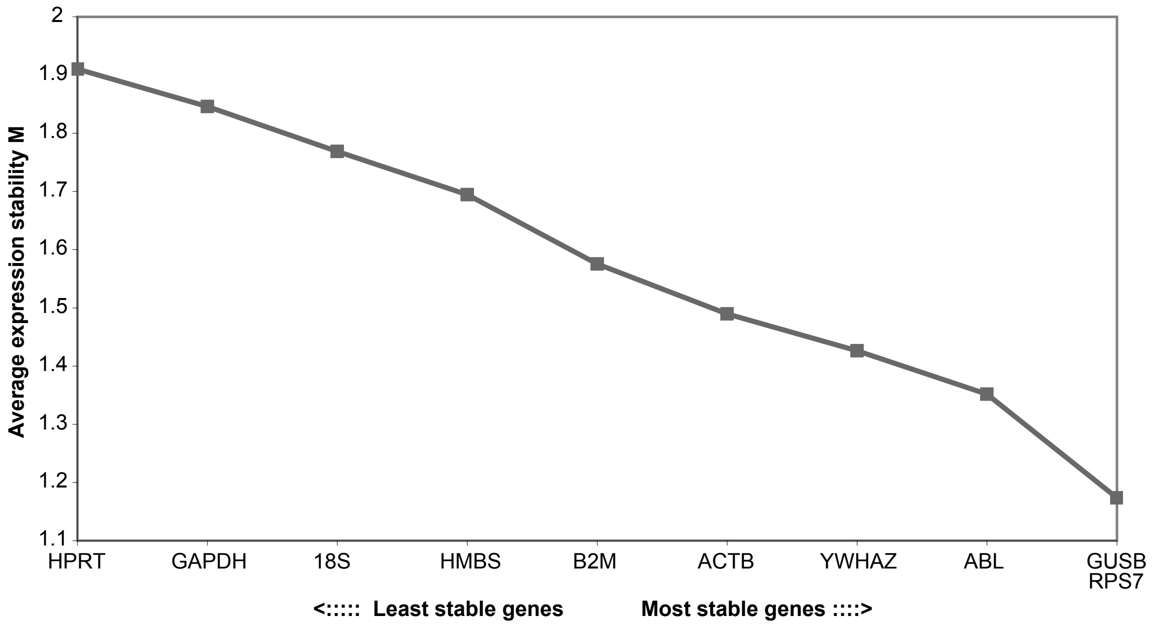

Determination of the optimal number of control genes for normalization

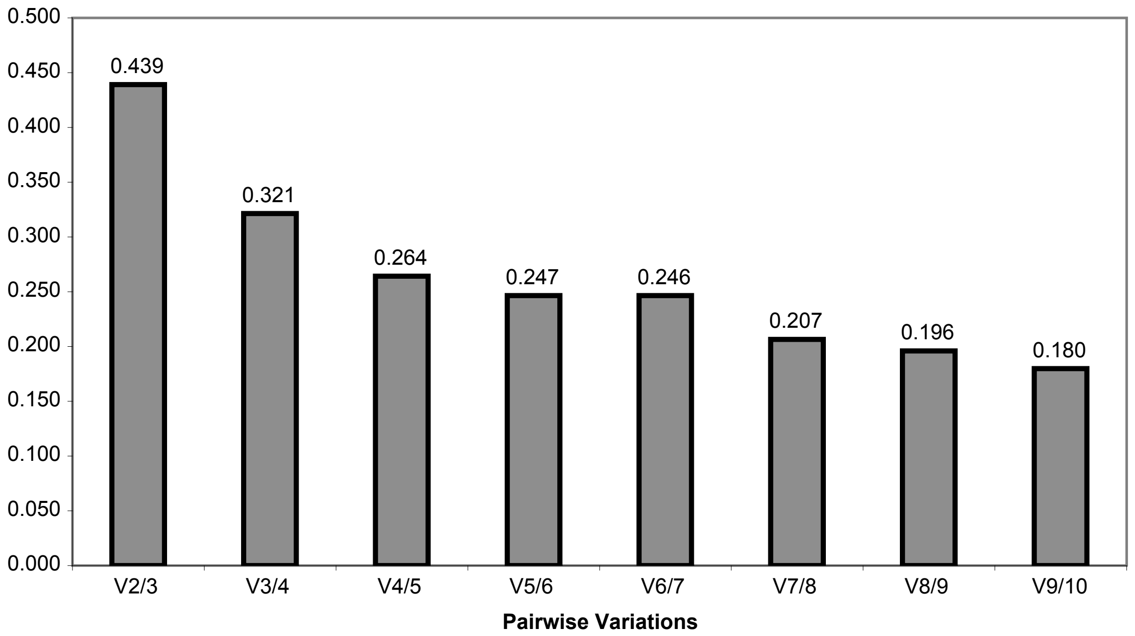

Additional File 2
r) geNorm output: Endocrine tissues

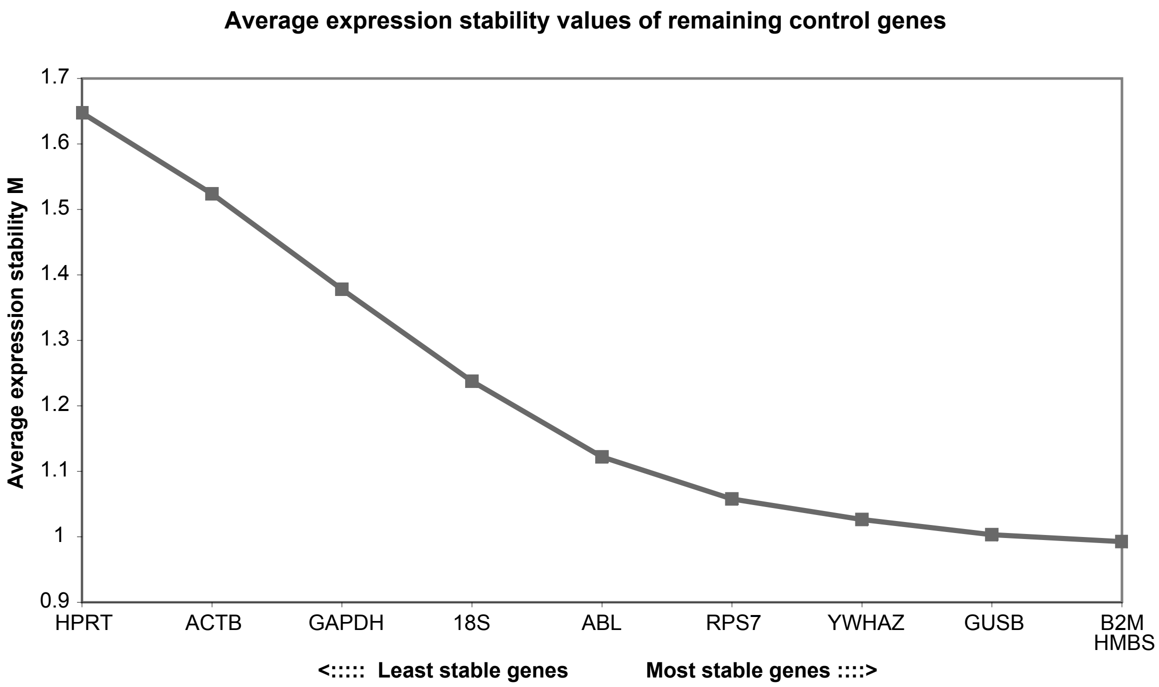

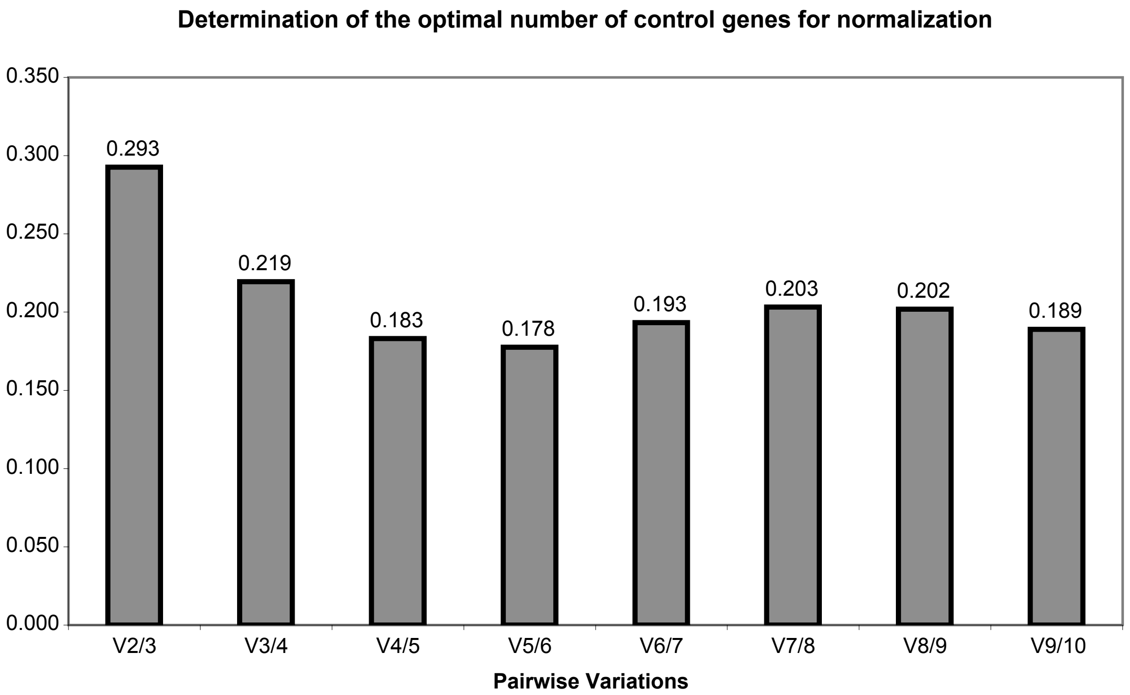

Additional File 2
s) geNorm output: Lymphoid tissues

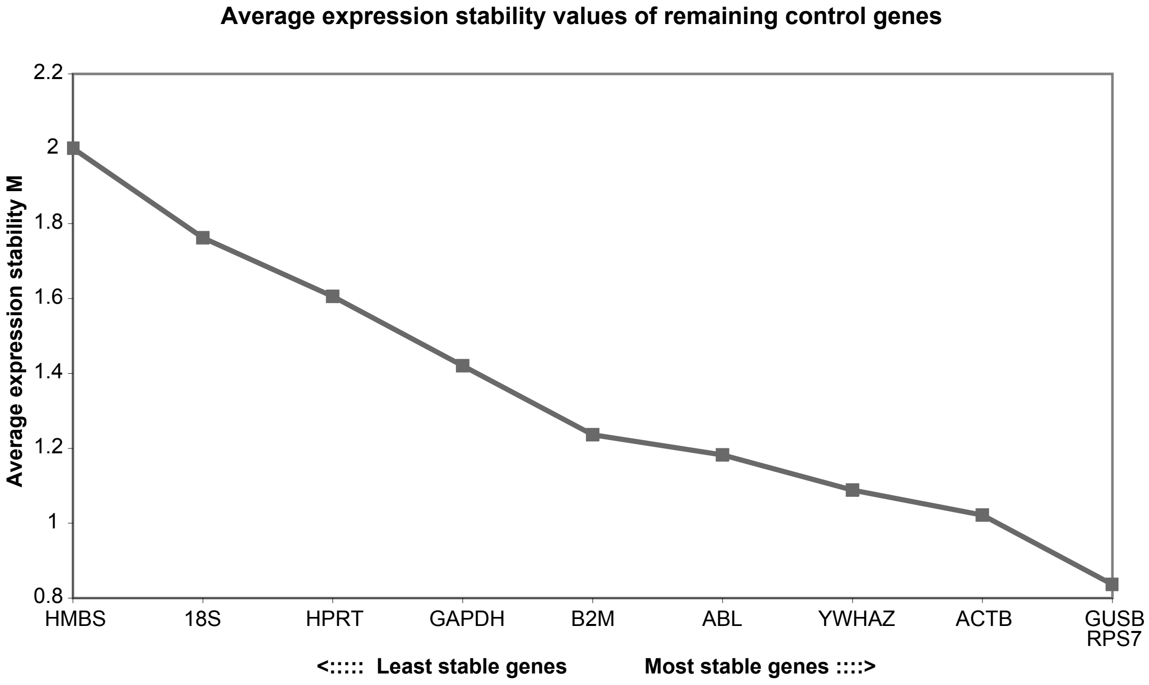

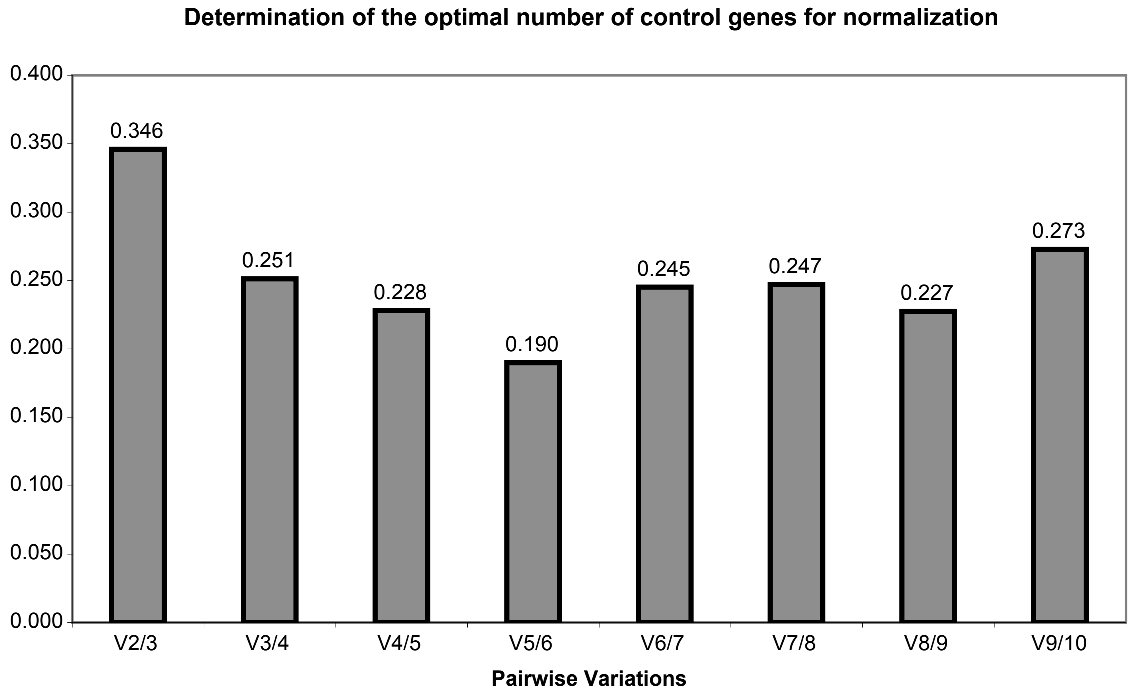

Additional File 2
t) geNorm output: Gastrointestinal tissues

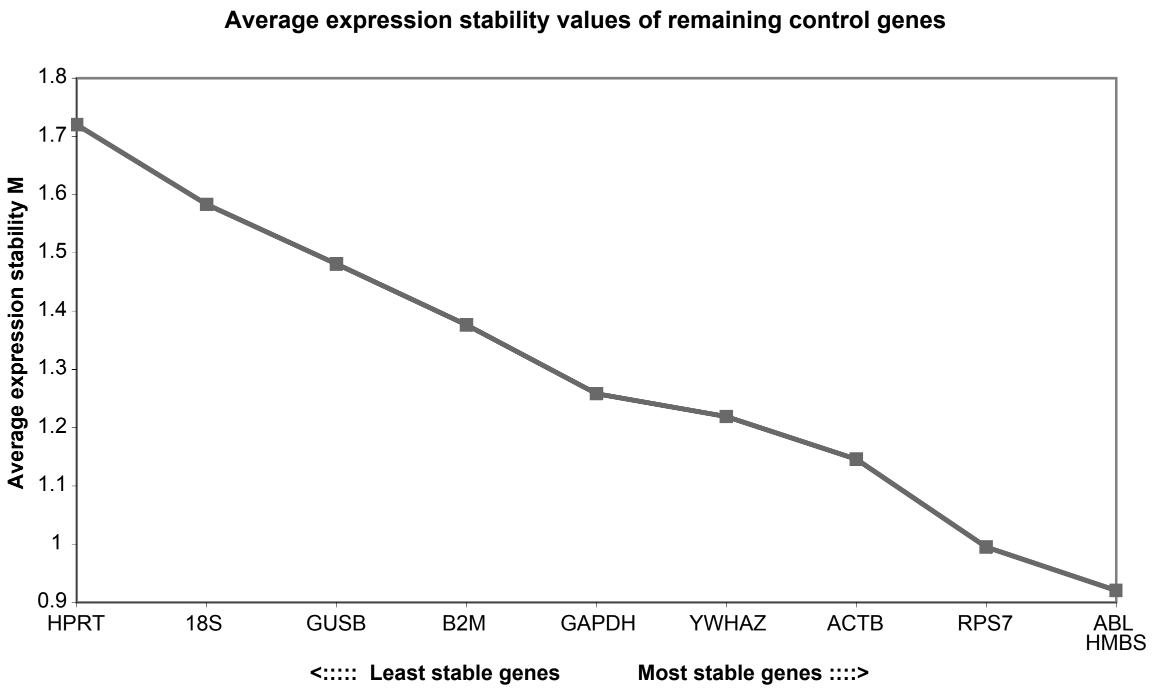

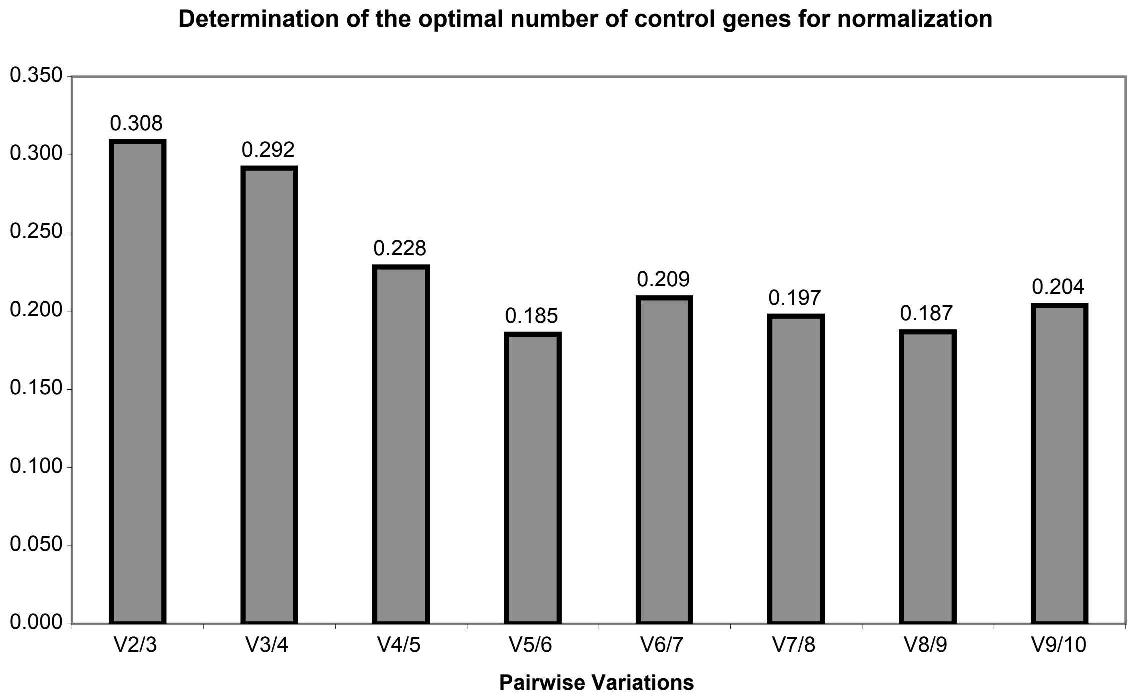

Supplement: Additional file 2 — geNorm output files. Average expression stability values calculated by the geNorm program for reference genes included in this study and graph showing the optimal number of reference genes for normalization. Output files are shown for a) adrenal gland, b) pancreas, c) parathyroid, d) thyroid, e) bone marrow, f) lymph node, g) spleen, h) parotid gland, i) duodenum, j) ileum, k) liver, l) kidney, m) myocardium, n) brain, o) blood, p) neoplastic tissues, q) all 14 healthy tissues combined, r) all endocrine tissues, s) all lymphoid tissues, and t) all gastrointestinal tissues. [file 1471-2199-10-106-S2.PDF]
